# *PICARA*, an Analytical Pipeline Providing Probabilistic Inference about *A Priori* Candidates Genes Underlying Genome-Wide Association QTL in Plants

**DOI:** 10.1371/journal.pone.0046596

**Published:** 2012-11-07

**Authors:** Charles Chen, Genevieve DeClerck, Feng Tian, William Spooner, Susan McCouch, Edward Buckler

**Affiliations:** 1 Department of Plant Breeding and Genetics, Cornell University, Ithaca, New York, United States of America; 2 National Maize Improvement Center, China Agriculture University, Beijing, China; 3 Eagle Genomics Ltd, Cambridge, United Kingdom; 4 Institute for Genomic Diversity, Cornell University, Ithaca, New York, United States of America; 5 U.S. Department of Agriculture-Agriculture Research Service (USDA-ARS), Robert W. Holley Center for Agriculture and Health, Ithaca, New York, United States of America; The Australian National University, Australia

## Abstract

*PICARA* is an analytical pipeline designed to systematically summarize observed SNP/trait associations identified by genome wide association studies (GWAS) and to identify candidate genes involved in the regulation of complex trait variation. The pipeline provides probabilistic inference about *a priori* candidate genes using integrated information derived from genome-wide association signals, gene homology, and curated gene sets embedded in pathway descriptions. In this paper, we demonstrate the performance of *PICARA* using data for flowering time variation in maize – a key trait for geographical and seasonal adaption of plants. Among 406 curated flowering time-related genes from *Arabidopsis*, we identify 61 orthologs in maize that are significantly enriched for GWAS SNP signals, including key regulators such as *FT* (*Flowering Locus T*) and *GI* (*GIGANTEA*), and genes centered in the *Arabidopsis* circadian pathway, including *TOC1* (*Timing of CAB Expression 1*) and *LHY* (*Late Elongated Hypocotyl*). In addition, we discover a regulatory feature that is characteristic of these *a priori* flowering time candidates in maize. This new probabilistic analytical pipeline helps researchers infer the functional significance of candidate genes associated with complex traits and helps guide future experiments by providing statistical support for gene candidates based on the integration of heterogeneous biological information.

## Introduction

Genome wide association studies (GWAS) have shed new light on the genetic basis of complex trait variation in diverse species [1]–[7], and contributed to our understanding of how natural allelic variation affects trait expression in diverse genetic backgrounds [8], [9]. A key objective of most association mapping studies is to identify quantitative trait loci (QTL) and ultimately to discover the genes or causal genetic variants that contribute to the observed phenotypic variation. In many species and populations, extended linkage equilibrium (LD) limits the resolution of GWAS, making it difficult to pinpoint which genes and casual variants are responsible for the observed phenotypic variation without additional genetic analysis [5], [10].

To facilitate the identification of genes underlying GWAS-QTLs in humans where controlled crosses and transgenic experiments are not feasible, a variety of statistical tools have been developed ([11]; ALIGATOR [12]; DAVID [13]; ConsensusPathDB [14] and ToppGene Suite [15]). These tools utilize enrichment statistics to search for candidate genes and functional polymorphisms associated with human disease. For example, genetic susceptibility alleles of CRC (Colorectal Cancer) were identified by the significant enrichment of GWAS SNPs in the *MAPK* (*Mitogen-Activated Protein Kinase*) signaling pathway [16]; also, the discovery of *TCF7L2* (*Transcription Factor 7-Like 2*), a human type 2 diabetes and cancer related genetic locus, was primarily driven by identifying the overrepresentation of significantly associated loci within 5 kb region of the target gene [17]. These examples in human medical research suggest that a systematic search for *a priori* candidate genes and rigorous methods for identifying underlying causal genetic variants would greatly enhance the power and efficiency of GWAS for the plant research community.

In plants, detailed studies of genetic mechanisms underlying diverse phenotypes have been undertaken using both forward and reverse genetics. In *Arabidopsis* and a few model crop species, these studies have productively utilized functional genomics populations developed in a limited number of genetic backgrounds using either chemical mutagenesis or transgenic activation of mobile elements [18]–[20]. Parallel studies have been undertaken using QTL mapping and positional cloning of natural alleles [21]–[25]. These studies are often complemented by expression analysis where targeted or global gene expression profiles are compared between a mutant and a wild type, in response to a particular stress or other treatment [26]–[30]. Such genetic information has been thoroughly reviewed and further organized using ontologies and controlled vocabulary in genome databases [31], [32]. Building on this foundation, it is essential to implement an analytical platform to facilitate the interpretation of GWAS results, and to systematically integrate all available genetic information pertaining to the same biological phenomena.

Recently, several large GWAS projects have been reported in plants [5], [6], [8], [9]. Co-localization of GWAS peaks (significant SNPs) and candidate genes associated with a common set of phenotypes have driven the interpretation of results. In most cases, GWAS SNPs located within 20–200 kb of *a priori* candidate genes have been specified as significantly associated with trait variation [5], [6], [8], [9]. Though the findings in these studies are encouraging, the lack of a dynamic and systematic approach for integrating relevant information across species and domains of biology warrants further investigation. Ultimately, the challenge is to associate naturally occurring variation (SNPs and indels) identified in significant GWAS regions of the genome with mechanisms that can explain and predict the observed phenotypic variation.

**Figure 1:**
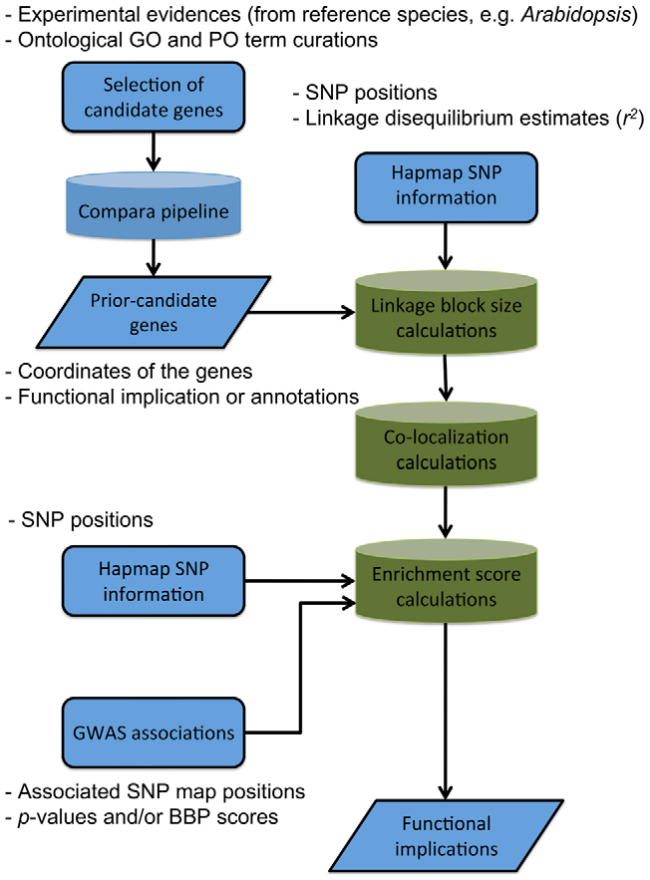
Enrichment analysis pipeline, and the data that is required in the procedure.

In this paper we present *PICARA*, a new probabilistic approach designed to efficiently search for and validate *a priori* candidates that are predicted to play a role in regulating quantitative variation and to integrate these candidates with information derived from GWAS signals (Figure 1). We address limitations of fixed window approaches that are either unlikely to capture distantly located trans-acting regulators, or falsely include potential candidates by overestimating the sizes of target haplotypes. This is accomplished by developing a dynamic algorithm that estimates linkage block size (or window size) around *a priori* candidate genes according to their local SNP distributions. The resulting linkage block size is then used to delineate the target haplotype containing SNP variants and potential *a priori* candidate genes of interest. With a Bayesian posterior probability that describes the likelihood of candidates co-localizing with significant GWAS association signals, *PICARA* generates a probabilistic inference for assessing *a priori* candidates with GWAS enrichment. In addition, the functional characteristics of *a priori* candidates are determined by a phylogeny-based multiple species gene homology search. Not only are the various LD patterns of a genome dynamically implemented in the pipeline, this new probabilistic inference also reckons the functional characteristics of candidates from distantly related species. The statistical support provided by *PICARA*'s approach can further assist in prioritizing candidate gene experiments based on reliable resource identification and integrated expert knowledge.

To demonstrate *PICARA*, we examine GWAS associations for days-to-silk flowering time variation in maize NAM (Nested Association Mapping) populations [33], provide a detailed description of the statistical framework in the *PICARA* pipeline, and then identify *a priori* candidates predicted to play regulatory roles mediating maize flowering time variation in the field. Our choice of flowering time in maize to illustrate the strength of this new tool is based on the importance of flowering time as a basic component of plant development, survival and fitness, the fact that a great deal is known about the genes and gene networks that mediate flowering time, and the fact that it is significantly correlated with many other traits of agronomic importance in crops [34].

Our probabilistic approach to investigate the functional implications of GWAS associations is designed to more efficiently utilize the wealth of knowledge that has been generated in reference species, including *Arabidopsis* and other economically and phylogenetically important plant species. Our case study of flowering time serves as an example to demonstrate how GWAS results focusing on natural variation of complex traits can be systematically integrated with information derived from basic genetic research. We highlight the value of information derived from functional characterization of genes based on mutagenesis and expressional profiling, and demonstrate how a deep understanding of basic biological processes can accelerate the application of this knowledge to the fields of plant breeding and agriculture. Our immediate goal is to promote the effective and systematic integration of heterogeneous biological data sets and to facilitate the formulation of readily testable hypotheses of interest to a broad range of life scientists.

## Results

### Maize days-to-silk GWAS

The number of days-to-silk (flowering time) varies by 32 days among NAM founder lines. A total of 613 associations responsible for days-to-silk variation were identified in the maize NAM populations based on a model re-sampling technique (RIMP ≥2, [6]). When applying a more stringent significance cut-off of RIMP ≥5, though the number of associated SNP was reduced to 229, a polygenic model with numerous associations is thus suggested. To summarize the overall distribution of associations across maize chromosomes, a Manhattan plot displaying all significant GWAS days-to-silk associations at RIMP ≥2 is shown in Figure S1.

**Figure 2:**
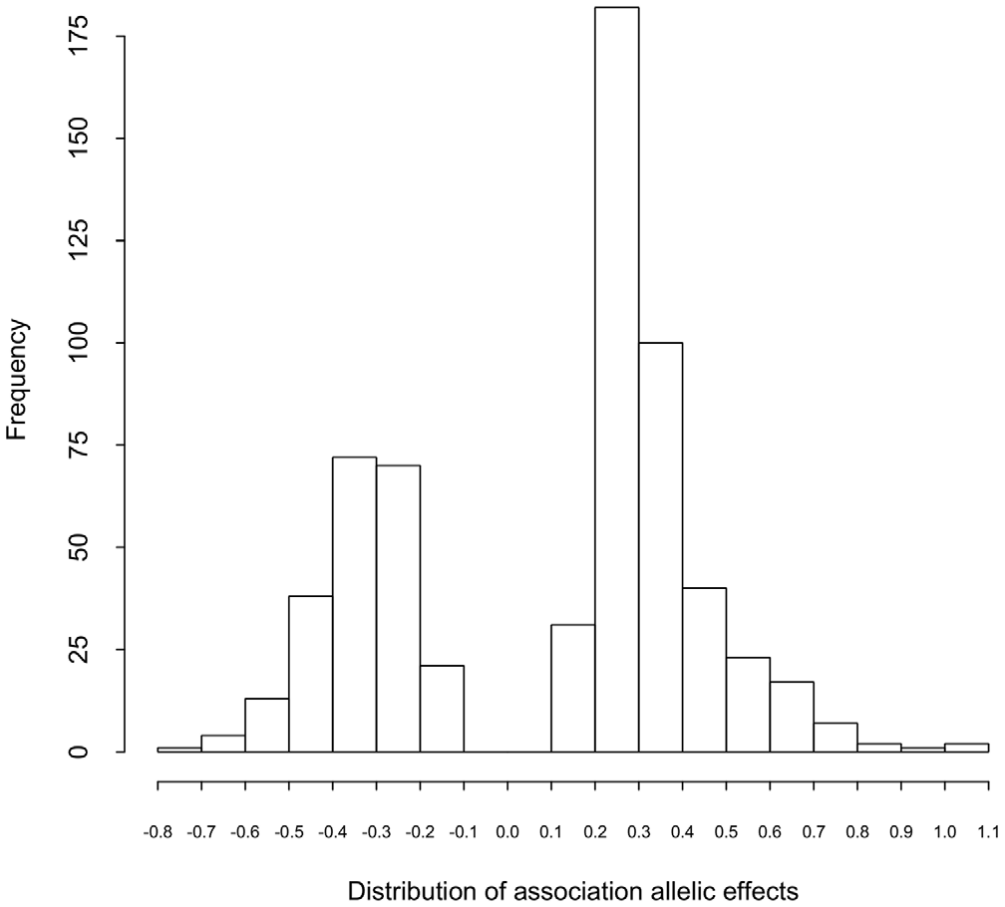
The distribution of allelic effects in maize days-to-silk associations. The unit of allelic effect is in day(s).

The SNP with the largest allelic effect was PZE10102590312 on chromosome 10, and it was associated a delay in flowering of 1.09 days compared to B73 (3.4% of overall variation); the SNP with the second largest effect, PZE10109543539, was also found on chromosome 10 (Table S2). With the majority of the allelic effects clustered within the range of 0.2 days, a polygenic model with many QTL associations is supported. The distribution of association effects is shown in Figure 2.

**Figure 3:**
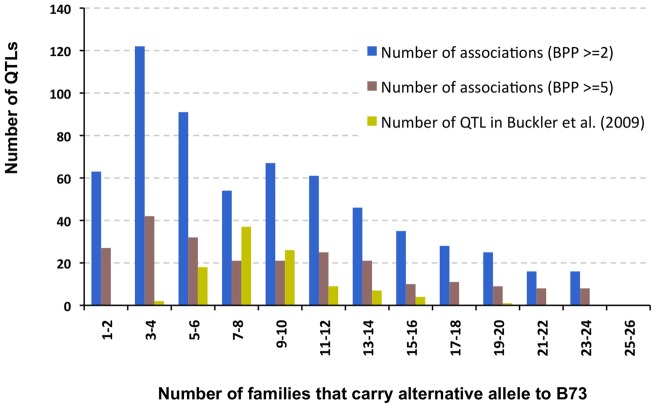
The distribution of association QTLs across NAM families. NAM GWAS associations identify a few cases of family specific QTL, while QTL found in previous joint-linkage analysis are mostly shared by 7 or 8 families.

In a joint linkage analysis, Buckler et al. [35] found that 2 QTLs showed an effect in only in three NAM families, while most of the maize flowering time QTLs were shared among multiple families; over 30% of QTLs were shared among seven or eight families. With a higher resolution of 1.6 million SNP markers, we identified a few cases of family-specific associations; 27 associations were shared between 2 families, while over 55% of associations were shared among at least eight families. In comparison to the findings in Buckler et al. [35], a pattern of a large number of associations and QTLs shared among multiple NAM families is illustrated in Figure 3.

**Figure 4:**
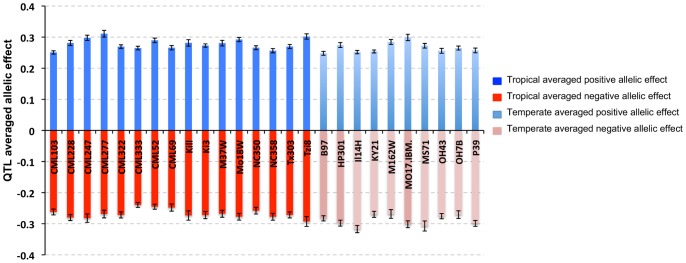
Averaged positive and negative allelic effects and their standard deviations in tropical versus temperate NAM populations.

Maize grows from the tropics into both northern and southern temperate zones, which can be differentiated with maize HapMap version 1 SNPs. We separated the NAM populations into tropical and temperate lines, according to Gore et al. [36], and found no difference in allelic effects controlling days-to-silk variation (Figure 4).

### Maize flowering time homologs

Across the genome as a whole, the Ensembl Compara pipeline identified 206,535 homology relationships between *Arabidopsis* and maize genes: 158,496 in the between-species-paralogous relationships, 33,931 in many-to-many orthologous relationships, 9,974 in one-to-many orthologous relationships, 4,073 one-to-one orthologs and 61 in the category of apparent one-to-one orthologous relationships.

**Figure 5:**
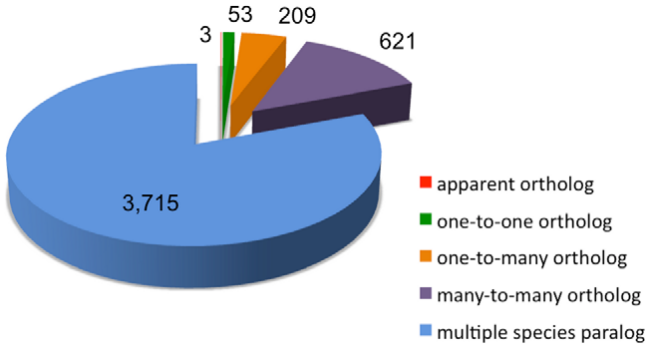
Maize flowering time related homologs, resulted from the comparison between *Arabidopsis* and maize genes by Compara pipeline.

When a curated set of 406 flowering time-related genes from *Arabidopsis* studies were used as the query (Table S1), 4,601 maize homologs were obtained from Compara results, including apparent orthologs (3 maize genes, on chromosomes 1, 4 and 5), one-to-one (53 maize genes), one-to-many (209 maize genes) and many-to-many orthologous relationships (621 maize genes) (Figure 5). Also, 3,715 maize genes were identified as multiple species paralogs, using *Arabidopsis* as the query. We then eliminated the duplicated maize homologs and finally compiled a set of 1,536 unique maize genes that were in any of the above described homology relationships with *Arabidopsis* flowering time candidates. Because of the conserved domains in genes from large gene families, like the MADS domain transcription factors *AGL1-AGL18*, the *AGAMOUS-like* gene family, or *PHYTOCHROME INTERACTING FACTOR 3-like* genes, *PIL2*, *PIL5*, *PIL6* and *PIL7*, over 60% of the *a priori* candidates (37 enriched maize homologs) were found to be in more than one homology relationship with multiple *Arabidopsis* flowering time candidates. In addition, as expected in the highly duplicated genome of maize [37], [38], the number of maize genes identified in paralogous homology relationships with *Arabidopsis* genes is also greater than those in orthologous homology relationships.

Important flowering time homologs were identified in maize. For example, *FT*, *FLOWERING TIME LOCUS T* (AT1G65480), a floral promoter situated in the center of *Arabidopsis* flowering time pathway as an integrator, is found to have 19 copies of between-species paralogs and 7 copies of many-to-many orthologs, distributed on almost all of the maize chromosomes. *LHY*, *LATE ELONGATE* (AT1G01060), has 13 homologs in the maize genome. Two important long-day pathway genes, *CO* (*CONSTANS*) and *GI* (*GIGANTEA*), are also duplicated in maize. We have identified 22 homologous copies of the *Arabidopsis CO* gene in maize, including the putative orthologue, GRMZM2G405368 on chromosome 2. There are, however, only two orthologous copies of *Arabidopsis GI* genes, GRMZM2G062262 and GRMZM2G107101, respectively, on maize chromosomes 3 and 8.

### Linkage block size estimations

**Figure 6:**
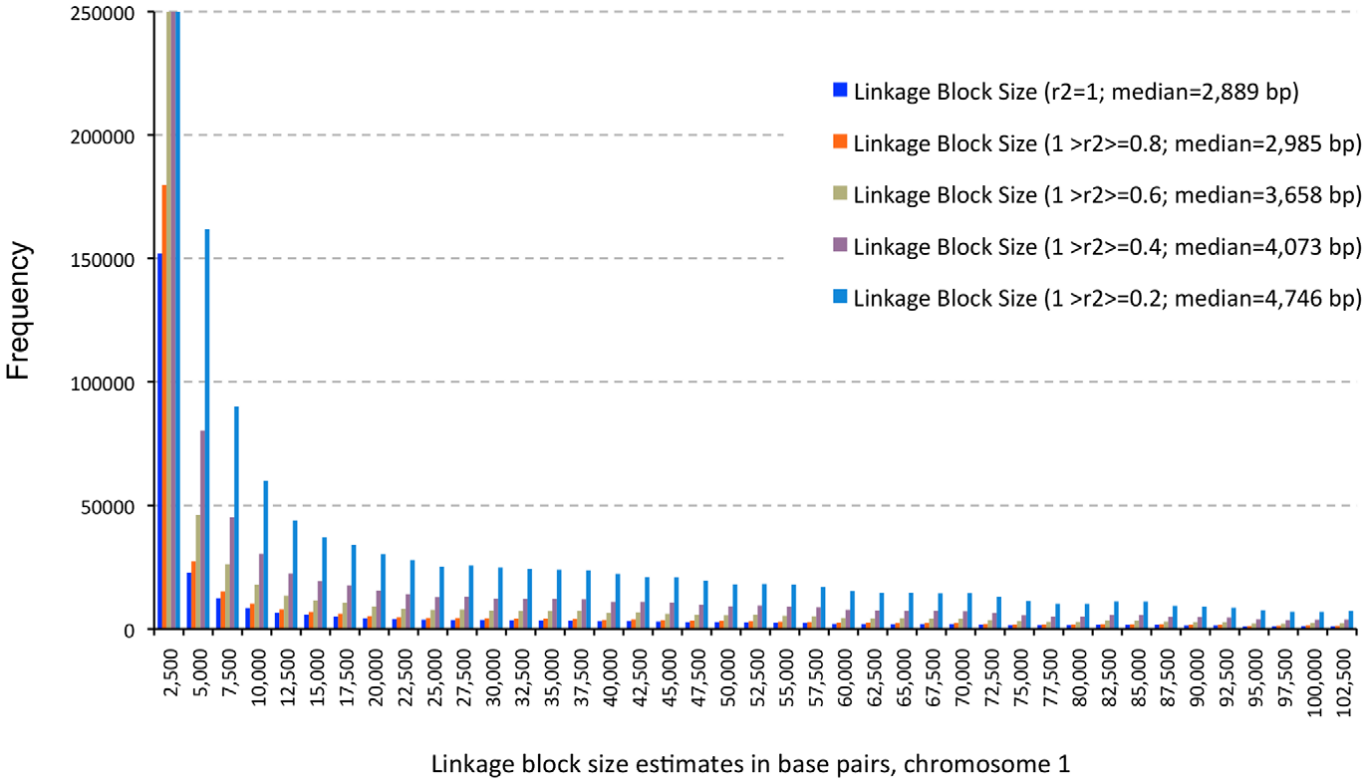
The comparison of estimated linkage block sizes with perfect linkage (*r^2^* = 1), high linkage (1>*r^2^*≥0.8), intermediate linkage (1>*r^2^*≥0.6 and 0.4) and low linkage (1>*r^2^*≥0.2).

Linkage block size estimates for chromosome 1 range from 2 bp to 855,908 bp, with a median of 3,055 bp and the 90% quantile estimated at 83,414 bp. Maize chromosome 4 has the largest linkage blocks (median  = 10,164 bp) while chromosome 8 has the smallest (median  = 2,294 bp). On the same chromosome, linkage block size estimates are similar where SNPs are in perfect linkage disequlibrium (LD) (*r^2^* = 1) or high LD (1>*r^2^*≥0.8), and linkage block size estimates start to increase at r^2^ values <0.8. Figure 6 shows an example of the distribution of linkage block sizes on chromosome 10, for linkage blocks with SNPs in perfect LD (*r^2^* = 1), in intermediate to low LD (1>*r^2^*≥0.4 and 1>*r^2^*≥0.2), in intermediate LD (1>*r^2^*≥0.6), and in high LD (1>*r^2^*≥0.8). In this study, only SNPs in perfect LD (*r^2^* = 1) were used to estimate linkage block sizes for a given *a priori* candidate gene. Though the LD block sizes do not vary dramatically for the first generation maize HapMap, the PIARA algorithm provides flexibility for recombination coefficients for use in analysis.

### Probabilistic model in maize flowering time GWAS association enrichments

With a curated set of 406 *Arabidopsis* flowering time-related genes, 61 maize homologs were significantly over-represented by days-to-silk flowering time GWAS associations, and 21 flowering time genes orthologous to curated gene sets from *Arabidopsis* studies were enriched with significant GWAS SNPs. The only enriched *a priori* candidate gene that is in one-to-one orthologous relationship with its *Arabidopsis* counterpart was a *PHP* (*Plant Homologous Parafibromin*) gene. This gene encoded a subunit in the *Paf1c* complex (RNA polymerase II associated complex), and the effect of the *cdc73* (Cell Division Cycle73) mutation on the *Arabidopsis PHP* gene was to strongly suppress the late flowering phenotype of *FRIGIDA*. This *Arabidopsis Paf1c* was also shown to participate in the modification of *FLC* (*Flowering Locus C*) chromatin and to affect *CO* (*CONSTANS*), *TSF* (*Twin Sister of FT*), *SOC1* (*Suppressor of Overexpression of CO*) and *AGL24* (*Agamous-like 24*) [39], suggesting a regulatory role of *PHP* gene in affecting flowering time pathway integrators.

**Figure 7:**
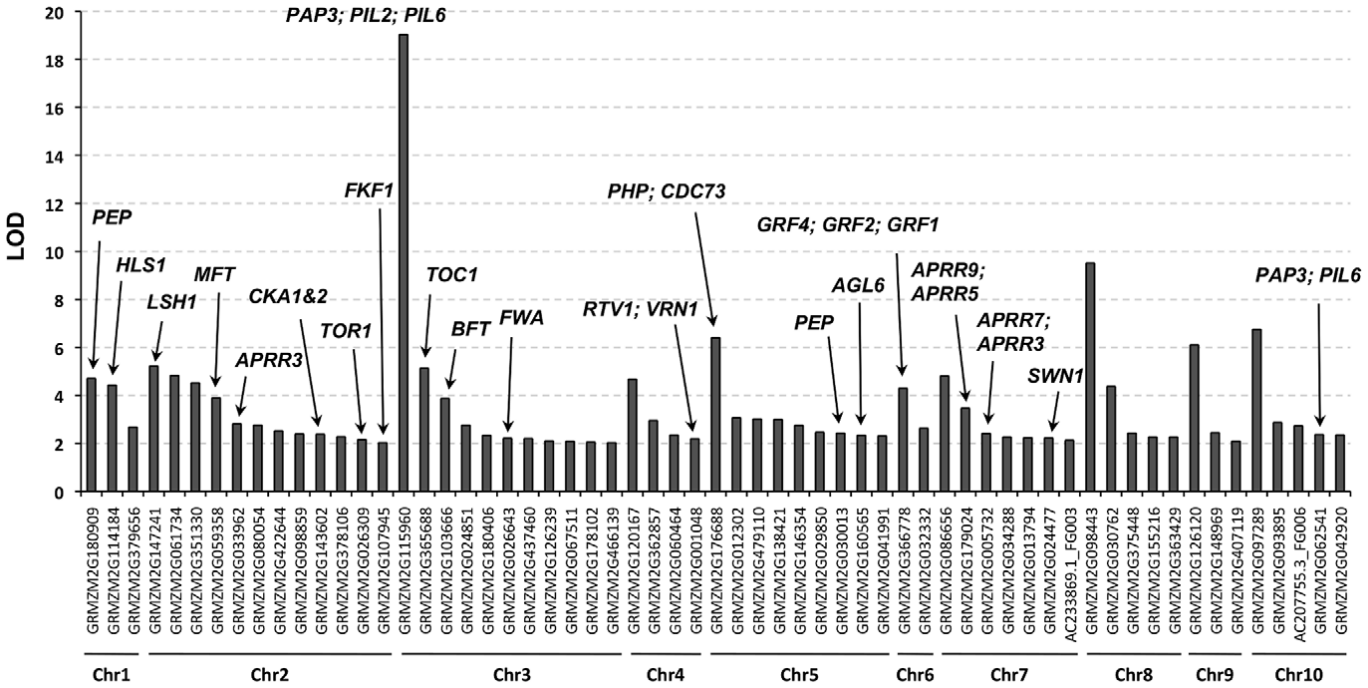
Maize flowering time priori candidate genes. Maize flowering time priori candidate genes are identified via enrichment of GWAS associations; and, the annotations are taken from their orthologous relationship to *Arabidopsis* genes.

**Figure 8:**
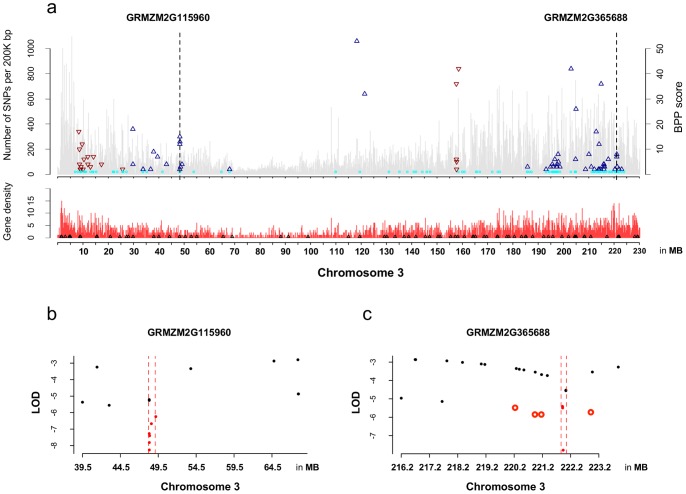
SNPs, GWAS associations, flowering time homologs and gene density on maize chromosome 3. In (a), the top panel shows the days-to-silk GWAS signals; blue triangles are the positive QTLs, the red are negative ones and the light blue ones at the bottom indicative of insignificant SNPs that did not pass RMIP test. SNP density along the chromosome is in grey bars in the background and significance level of RIMP scores is in the axis on the left. The gene density, calculated from the number of maize gene in every 200K bp, are in the lower panel, while the black triangles on the bottom of the density distribution mark the positions of maize flowering time homologs. Two dashed vertical lines indicate the positions of the examples in two top enriched flowering time maize homologs. (b) The enrichment of maize flowering time priori candidate: GRMZM2G115960. In this case, the co-localizing significant QTLs found in GWAS are all within the linkage block of the *a priori* candidate. (c) The enrichment of maize flowering time priori candidate: GRMZM2G365688. Three (solid red dots) of 6 significantly QTLs reside within linkage block of *a priori* candidate; the 3 unfilled red dots outside of the dashed red lines are significant, but unlinked, GWAS associations, while black dots being maize Hapmap 1 SNPs.

Figure 7 shows all maize flowering time *a priori* candidate genes, and the annotations of maize *a priori* candidates derived from *Arabidopsis* orthologs, when the orthologous relationship can be identified. The highest count of association signals in a single linkage block corresponds to the maize candidate gene, GRMZM2G115960 on chromosome 3, a duplicated homolog of transcription factor *PIF3* (*Phytochrome Interacting Factor 3*) that interacts with *PHYA* (*Phytochtome A*) and *PHYB* (*Phytochrome B*) in the *Arabidopsis* circadian pathway [40], [41]. With the linkage block size estimated at 141,762 bp, six GWAS associations were found significantly co-localizing with maize GRMZM2G115960; among them, two highly significant associations were found at 396 bp (in the 5′ UTR) and 1,268 bp downstream of the gene (with RIMP count of 15 and 12, respectively) (Figure 8(b)).

Chromosome 2 showed the greatest number of associations and the largest number of candidate genes enriched for significant SNPs. Twelve flowering time-related *a priori* candidate genes were enriched for significant associations on this chromosome. Chromosome 6 had the least number of associations (39 significant associations with RIMP >1), as well as the least number of enriched flowering time *a priori* candidates (two genes). Two of the most significant SNPs (PZE10102590312 associated with an allelic effect  = 1.09 days to silking and PZE10109543539 associated with 1.06 days) were found on chromosome 10, but were not associated with any flowering time *a priori* candidate genes, both being at least 1 million base pairs away from the closest potential *a priori* candidate. These are likely to represent novel loci and warrant further investigation.

A few major flowering time pathway integrators were found in association with significant SNPs using our analytical pipeline. These include a maize homolog of the *Arabidopsis* FT (*Flowering Locus T*) gene, GRMZM2G103666, a *CONSTANS* homolog, GRMZM2G2G041991, a homologous *CONSTANS*-like gene, GRMZM2G041991, and a homologue of the *Arabidopsis STO* (*Salt Tolerance Protein*) gene that interacts with the *COP1* (*Constitutive Photomorphogenic 1*) gene in light signaling, GRMZM2G422644, as well as several genes involved in floral transition, such as the maize homolog of *LHY*, GRMZM2G029850.

In addition, we found a majority of *a priori* candidate genes is involved in the regulation of the circadian pathway. Examples include the maize gene, GRMZM2G365688 on chromosome 3, an ortholog of *TOC1* (*Timing of CAB Expression 1*) (Figure 7), maize GRMZM2G080054, a *PIF* and *PIL* (*Phytochrome Interaction Factor Like*) homolog that is involved in photo-morphogenesis in *Arabidopsis*, maize GRMZM2G479110, a homolog of the *Arabidopsis* phyto-clock-like genes, *PCL1* (*Phytoclock 1*) and *LUX* (*LUX ARRHYTHMO*). It is noteworthy that *PCL1* and *LUX* promoters are required in the regulation of *TOC1*, *CCA1* (*Circadian Clock Associated 1*) and *LHY*. Details of enriched flowering time *a priori* candidate genes in maize are listed in Table S3, along with annotation evidence taken from *Arabidopsis* experiments.

To compare the strength of *PICARA* with the synteny comparison, we conduct a flowering time QTL search using the Gramene QTL database [42]. To show an example, in the chromosome 7 region containing the maize *CIB1* (*Cryptochrome-Interacting Basic-Helix-Loop-Helix*) homolog, there are no previously defined maize QTL. The nearest days-to-silk QTL (at 63.7 cM on chromosome 7, associated with marker m798, as reported in Buckler et al. [35] is about 1 MB upstream from the *PICARA* identified *CIB1* maize homolog. We then complete a synteny search using 685 flowering time QTLs from rice studies (search includes the trait names: days to heading, days to flowering and days to maturity). With the CMap tool [43], we identify the corresponding syntenic region, defined by the location of m798 on the maize 2008 NAM map, which lies in the region flanking RM242 and RM108 on rice chromosome 9 (QTL Accession ID: AQGP041 on Gramene database) [44], [45]. The possible flowering time-related QTL from the comparative synteny analysis was found about 10 MB away from the position of the maize *CIB1* homolog.

In addition to examining candidate genes associated with flowering time GWAS signals, we also re-analyzed upper leaf angle GWAS results and compared the output of *PICARA*, with the list of potential *a priori* candidates provided in [6] (Supplementary Table 7). Tian *et* *al*. [6] identified two candidate genes with significant enrichments of GWAS SNPS for upper leaf angle, *lg1* (*liguless1*) and *lg2* (*liguleless2*), on chromosomes 2 and 3, respectively. With our new probabilistic approach, in addition to *lg1* and *lg2*, we also found significant support for GWAS SNP enrichment at *lg4* (*liguleless4*) on chromosome 8, which had been considered insignificant based on the original cumulative RIMP count [6]. The linkage block containing maize *lg4* (GRMZM2G094241) is about 78 KB long, slightly larger than reported previously, and contains a significant SNP association (RIMP  = 3) at 41 KB upstream of the gene.

To further validate results from *PICARA*, we first excluded the 1,539 maize flowering time-related candidates from the release 5a filtered gene set of the maize genome [38], and we generated 100 pseudo-candidate gene sets of 1,539 randomly selected maize genes for each set and searched for days-to-silk enrichment via *PICARA*. The average LOD score from these 100 pseudo-candidate sets was <0.01.

### Gene ontology (GO) analysis

**Figure 9:**
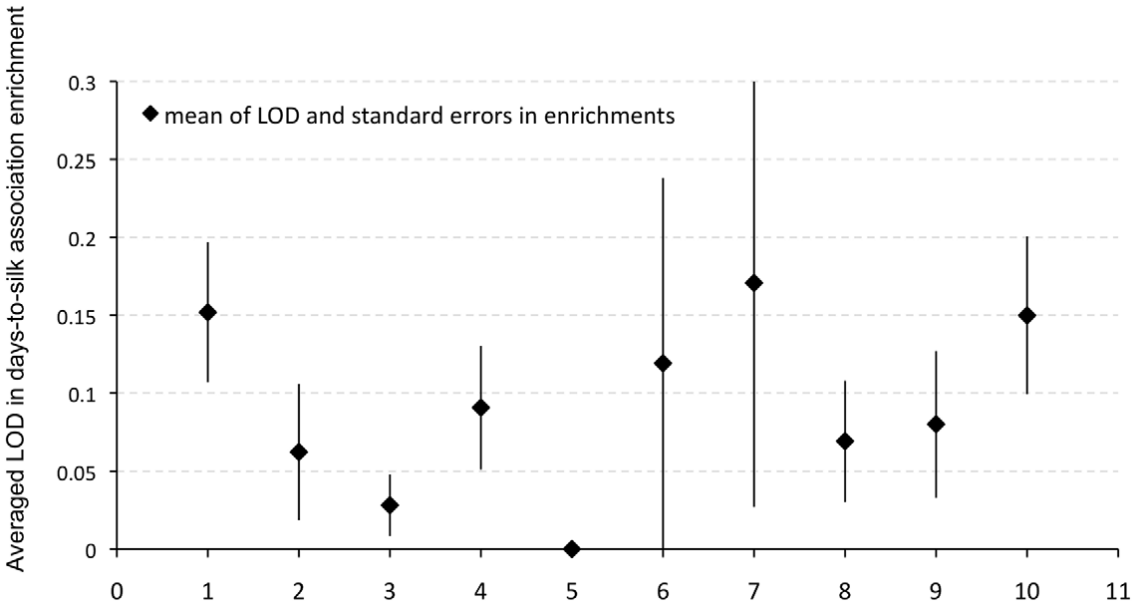
Flowering time variation association enrichment LOD scores comparison in different curation categories. 1: maize flowering time orthologs; 2: maize leaf genes (in Feng *et* *al*. 2010); 3: maize miRNA target leaf genes; 4: biosynthetic process (GO:0009058); 5: developmental process (GO:0032502); 6: enzyme regulator activity (GO:0030234); 7: growth (GO:0040007); 8: negative regulation of response to stimulus (GO:0048585); 9: positive regulation of response to stimulus (GO:0050729) and 10: transcription regulator activity (GO:0030528). GO terms in 6 and 10 are from molecular function GO terms, while the rest of the GO terms come from biological process.

Using a GO-annotated maize genome as a background reference, we identified 39 GO terms that were over-represented by our maize flowering time *a priori* candidate genes; of these, 32 were associated with biological process, 6 with molecular function and 1 with cellular component (Table S4). As for molecular function (GO:0005554), the *PICARA*-identified maize flowering time *a priori* candidate genes show an overrepresentation in GO:0030528, GO:0043565, GO:0003677, GO:0003676, GO:0003700 and GO:0005488, all classified using transcriptional regulatory activity and sequence-specific DNA binding parent terms. In the category ‘biological process’ (GO:0008150), a number of parent GO terms, such as GO:0065007 (regulation of transcription), GO:0019219 (regulation of nucleobase, nucleotide, and nucleic acid metabolic process), GO:0051171 (regulation of nitrogen compound metabolic process) and GO:0006350 (transcription) and GO:0009889 (regulation of biosynthetic process), were also highlighted (Figure 9, Table S4).

While the list of potential *a priori* candidate genes can be created based on other knowledge, such as BLAST searches for sequence similarities, pathway analyses or ontology terms, our list of flowering time *a priori* candidates was assembled based on genes that were experimentally supported from *Arabidopsis* studies. To summarize the effectiveness of our enrichment analysis in targeting *a priori* candidates, we compiled a similar list of maize orthologs to *Arabidopsis* candidate genes identified in nine other GO categories, and compared the level of GWAS SNP enrichment across these categories in our flowering time study (*Arabidopsis* gene IDs and ontology terms are listed in Table S5). Figure 9 summarizes the results of this comparison, and while most categories show negligible levels of enrichment, we do observe a close relationship of days-to-silk GWAS associations with genes involved in plant growth (GO:0040007) and, again, with transcription regulator activity (GO:0030528). The high average LOD score found in the growth (GO:0040007) category is due to the overrepresentation of GWAS associations in GRMZM2G117935, an ortholog of the *Arabidopsis SAL1* gene (*Supernumerary Aleurone Layer 1*) that is known to play a key role in seed development in *Arabidopsis* [46] and barley [47].

## Discussion

With this paper, we present *PICARA*, a new analytical pipeline that can systematically and dynamically integrate genomic knowledge derived from heterogeneous sources and provide a probabilistic reference of functional characteristics for *a priori* candidates. The probabilistic inference presented by *PICARA* also provides a platform that accounts for the uncertainty in co-localizing GWAS signals with potential gene candidates and functions inferred from a distantly related model system. In the comparison with other enrichment analyses [8], [48], [49], the flexibility and strength of *PICARA* are enhanced by the implementation of a method for calculating local LD structure, as well as a procedure for weighting significance levels derived from GWAS associations. Calculated from SNPs that are locally distributed around potential candidate genes, *PICARA* allows the variation of a genome's linkage structure to be analyzed locally, instead of using an arbitrary, fixed window size as in previous methods. In our flowering time example, recombination ratios vary dramatically along the maize chromosomes, as calculated from first generation maize Hapmap data (roughly one variant every 44 bp, [36]) Assuming causative polymorphisms would co-locate in high LD regions with *a priori* candidate genes, as shown in Figure 8(c), our probabilistic approach is able to determine the linkage block size locally and then successfully eliminates unlinked GWAS signals that might be improperly included in other fixed window analyses.

In addition, rather than only focus on the counts of significant associations, *PICARA*'s weighting procedures incorporate the strength of associations to determine significance levels in its re-sampling statistics of GWAS. In other words, the weighting procedure carried out in *PICARA* represents a measure of the magnitude of GWAS effects. We use a posterior probability that describes whether or not an *a priori* candidate gene co-localizes with a significant magnitude of GWAS enrichment signals. *PICARA*'s probabilistic inference assisted in finding the SNP enrichment for GRMZM2G37956 on maize chromosome 1, where there are only 75 SNPs in the region and the high linkage disequilibrium structure is less perceptible because of a relatively low level of marker resolution.

Through comparative QTL analyses, convergent selection was suggested to influence independent domestication processes of several closely related cereal crops, where variation for agronomically important phenotypes, such as flowering time, is thought to be governed by a limited number of common QTLs that are detectable due to conserved synteny even after millions of years of divergence [50]. This conservation of genomic colinearity among evolutionarily related species has enabled us to detect functional alternations associated with particular marker haplotypes [51], [52], as well as to predict and annotate mapped genes [53]. With the most comprehensive comparative genomic data coming from the grass family, synteny analysis tool such as CMap have been useful in translating genomic information among family member species [43]. However, the difficulty in identifying regions of co-linearity among highly divergent species underscores the limitations of synteny comparisons [54]. As the most in-depth genetic knowledge in plant research can be found in *Arabidopsis* systems, *PICARA* has exploited functional evidence and orthologous relationships in maize derived from the distantly related *Arabidopsis* study system. This work has been greatly facilitated by the development of a comparative genomics tool, namely, Ensembl COMPARA comparative genomics pipeline [55], allowing us to readily explore the genetic functionality that characterizes the underlying genetic architecture of quantitative trait variations in important cereal crops as outlined in the example for AC233869.1_FG0003, where we identify a maize ortholog of the *CIB1* gene (in Results and Table S3).

Together with the identification of the maize *CIB1* homolog, *PICARA*'s probabilistic inference also provides a confidence measure in the functional interpretation of GWAS associations: in the case of the maize *CIB1* homolog, where SNP polymorphism is found tightly linked with an *a priori* candidate (the linkage block size is estimated as small as 4,335 base pairs, Table S3), the GWAS data immediately suggests a possible causative role of the maize *CIB1* homolog. This functional interpretation of genetic architecture of flowering time variation, however, would have been missed by comparative synteny analysis, owing to its low resolution and lack of comparability with a distantly related study system.

In this paper, we adopt functional inferences derived from the foundation of molecular biology: a protein's sequence determines its structure, which in turn determines how the protein functions. These sequence-structure-function dependencies allow us to assess the functional similarity of maize genes via sequence and structure similarity with distantly related reference systems. Although the likelihood that orthologs and paralogs retain functional similarity is still an under-studied area, in a large comparison of 284,459 pairwise structure-based alignments of 12,634 unique domains from a protein database, Peterson *et* *al*. [56] reported that orthologs with high sequence similarity (>70% target-template sequence identity) are generally considered to retain function, and share greater structural similarity with a reference protein than are paralogs. Due to the redundancy inherent in gene duplication, functional characteristics of paralogs are often relaxed, allowing for more significant sequence changes to alter their structure, and in turn resulting in a higher probability of functional diversification among paralogs [57], [58].

When we focus only on the 21 *a priori* candidate genes that were orthologous to curated gene sets from *Arabidopsis* (Figure 7), it is notable that nearly half of them were involved in the regulation of light signaling pathways, including the maize orthologous copy of *TOC1*, genes in the *PIL* gene family as well as the only one-to-one orthologous *CDC73*. Single gene analysis may miss important unifying biological impacts that can be more difficult to interpret, especially when each allelic substitution governs a minor fraction of the observable quantitative variation. Using a GO-annotated maize genome as a background reference, maize *a priori* candidate genes can be summarized to be largely involved in transcriptional regulatory activity and sequence-specific DNA binding (parent terms: GO:0008150), and these same regulatory terms are also highlighted by *a priori* candidate genes associated with biological process. This emergent property of *a priori* candidate genes identified by *PICARA* (Figure 9, Table S4), namely the regulatory nature of maize flowering time GWAS associations may also provide insight into the evolution of the highly polygenic genetic architecture of maize flowering time variation. Compared to the GWAS findings in *Arabidopsis* flowering time studies, where nearly 50% of observed variation was explained by 12 major QTLs and their interaction with the growing environment [59], maize flowering time variation is determined by many more alleles with individually small effects (235 highly significant associations and the strongest allele effect is no larger than 3.4% in Table 2S).

Theoretically, the accumulation of small mutations that lead populations toward the optimum in a phenotypic hyperspace is the essence of Fisher's genetic adaptation model [60], whereas Motoo Kimura has pointed out that in Fisher's geometrical model, mutations with intermediate effect would have a higher probability of becoming fixed [61]. In a simulation study aiming to revise Fisher's infinitesimal model, Orr [62] further argued that the absolute distribution of fixed mutational effects was exponential, such that a small number of sizable mutations occur early while the population mean is distant from the adaptive optimum, and then a large number of small mutations arrive as populations progress toward adaptive peaks. Given a relatively simple genome, a greater degree of phenotypic differentiation (from 11 to 117 days to flowering) [59] and a deeper coalescence history among *Arabidopsis* strains than among maize varieties (*A. thaliana* and its close relative *A. lyrata* separated about 3.0–5.8 mya) [63], a larger distance between adaptive valleys and peaks in the fitness landscape created by *Arabidopsis* accessions can then be expected, and that consequently allows mutations with large effects to be favorable.

As for maize, it has a larger genome, and was domesticated relatively recently (6,000 to 10,000 years ago, [64]). Though a large amount of genetic diversity has been reported in maize inbred lines [35], maize NAM populations may establish a wider distribution of inter-connected demes in a fitness landscape, with relatively lower adaptive peaks. As a result, fixation of mutations with large effects is theoretically unlikely because they tend to overshoot the optimum from the adaptive space and hence they become deleterious. Small and regulatory mutations are thus favored in the maize adaptive process. Large numbers of mutations of small magnitude may also reduce the opportunity for unpredictable and sub-optimal epistasis to occur.

## Materials and Methods

### Maize NAM-GWAS on days-to-silk flowering time variation

The Maize NAM (nested association mapping) population consists of 5,000 recombinant inbred lines derived from each of the 25 crosses between diverse parents and B73 maize varieties [65]. In this paper, we also included the public maize intermated IBM population, MO17xB73, totaled 26 families in the flowering time GWAS analysis. Details about the design of maize NAM can be seen in Yu *et* *al*. [33] and Buckler *et* *al*. [35]. Maize NAM RIL lines and parents were then genotyped with the 1.6 million SNPs from Maize HapMapV1 [36].

In flowering time phenotyping, details for the days-to-silk (female flowering) phenotypes, the best linear unbiased predictors (BLUPs) of flowering time phenotypes for all NAM lines and the cross means are available in the supporting material of Buckler *et* *al*. [35]. Genotypes and phenotypes used in this research are all available and searchable through Panzea database (www.panzea.org), home of genetic architecture of maize and teosinte.

The complete details of missing genotype imputations, joint linkage model by stepwise regressions, GWAS association analyses with correction of population sub-structure and relatedness among NAM RIL lines and RIMP significance test of GWAS association can be seen in in Buckler *et* *al*. [35] and Tian *et* *al*. [6].

### Flowering time gene curation

The identification of *Arabidopsis* genes responsible for phenotypic variations via TAIR database (www.arabidopsis.org) has become a convenient and important resource for gene discovery. In addition to genes documented on the TAIR database, we also assembled curated information from the published literatures. In total, for our analysis, 406 *Arabidopsis* candidate genes are curated with evidence supported from enzyme and binding assays, functional, genetic and hybrid interactions, expression profiling, and mutagenesis experiments. The gene IDs, position information, annotation notes and references for the candidates are listed in Table S1.

### Compara homologs search

Using the reference list of 406 curated *Arabidopsis* genes, we performed a global search on maize AGPv1 filter gene set based on coverage in homology relationships, duplication consistency score and consistency of a multiple species phylogenetic framework, provided by Ensembl Compara comparative genomic pipeline [55]. Input for Compara pipeline consists of the longest translation for each gene locus, filtered for transponsons and other low-confidence genes from available whole genome sequences; clustering was performed by all-versus-all BLASTP followed by the extraction of genes linked either by best reciprocal BLAST, or BLAST score ratio larger than the threshold of 0.33. For each resulting cluster, we conducted multiple alignment based on protein sequences, inferred the evolutionary relationship by reconciling gene trees with the established species tree topology and determine ortholog/paralog calling based on the internal nodes annotated to distinguish speciation/duplication events of a rooted phylogeny [38], [55].

This orthology pipeline that we used in searching flowering time homologs in maize, rice or related species based on curated *Arabidopsis* flowering time candidate genes is currently hosted both on Gramene database (www.gramene.org) and Maizesequence (www.maizesequence.org). The resulting list of maize homologs is then used as the potential *a priori* candidate genes for later analyses.

### Linkage disequilibrium (LD) calculations

All 1.6 millions of SNP loci from maize first generation hapmap were first filtered for MAF (minimum allele frequency) larger than or equal to 0.05. Pair-wise linkage disequilibria of the filtered SNPs are estimated by the correlation coefficients (*r^2^*) between alleles at any given two polymorphic SNP loci [66]. SNP MAF filtering and LD calculations were conducted using TASSEL [67].

### Linkage block size estimations

Though average LD (linkage disequilibrium) in maize declines rapidly with distance [68], patterns of linkage disequilibria (LD) in the maize genome vary greatly within and among chromosomes [69]. The global estimate of linkage block size of a chromosome was calculated by taking the median of all distances in base pairs (bp) between all filtered SNP pairs that are in a perfect LD (*r^2^* = 1) with each other.

To preserve such LD structure in the analysis, we also estimated linkage block sizes for every *a priori* candidate gene, using the local LD information from the SNP loci that are located within and around the genes. Suppose that the *G_k_* denotes the selected list of potential a priori candidate genes (*k* = 1, 2, 3,… k) on a chromosome, and *S_i_* (*i* = 1,2,3,… i) is the polymorphic SNPs on the same chromosome. For each of the a priori candidate genes (*k*-th a priori candidate on a chromosome), a list of SNP loci (*S_k_*), including the SNP loci in the gene as well as the loci flanking it, is generated based on the map physical coordinates. A priori candidate gene block sizes are defined by the distances (*d_ik_*) between the SNP loci in (*S_i_*; 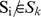) *that* appear to be in a perfect LD (*r^2^* = 1.0) with (*S_k_*), from both upstream and downstream.
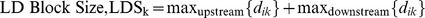



For the prior candidate genes that are located in SNP poor regions, where we cannot generate (*S_k_*) for the a priori candidate, we use the genome-wide estimates to compute the linkage block size. This whole genome-wide estimation would likely lead to an overestimate of the number of associations for such a priori candidate genes. We further filtered association signals with the co-localization analysis.

### Co-localization of *a priori* candidates and GWAS association signals with RIMP score weighting

For every prior candidate gene, an empirical cumulative probability distribution was computed, taking the physical distances from the center of the gene (*G_k_*) to all the SNPs (*S_i_*) on the same chromosome. Let Q*_m_* (*m* = 1, 2, 3,… *m*) be the GWAS associations identified for a given trait on a chromosome. Given the distance between a potential a priori candidate gene to a significant association signal, the conditional probability (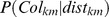) of the gene (*G_k_*) and the association (*Q_m_*) co-localizing can then be generated from the function of the empirical cumulative probability function of the a priori candidate.

In general, data from association studies screen thousands of SNP loci and most studies have corrected the significance levels with Bonferroni correction or false discovery rate procedures. In order not to overemphasize association signals while still retaining reliable information of the importance of hypotheses, we describe a procedure of weighting the posterior probability of co-localization with the ranking of significance levels p-values.

Treating all insignificant SNP loci (that did not pass the cutoff threshold) the same, we ordered GWAS association p-values by *P_i_* = (*p_0_*, *p_1_*, *p_2_*, …*p_i_*), where *p_1_*<*p*_2_<…*p_i_*; and, *p_0_* denotes the insignificant p-values for all insignificant SNP loci, and then ranked them by *R_i_* = (1, 2, … *r_i_*). The weights are: 
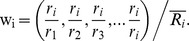


For flowering time associations in maize, the weights of association signals were given from the order of counts computed with the procedure of bootstrap posterior probability, where RIMP ≥2 (re-sampling model inclusion probability) indicates the significance of GWAS signals. For other cases, p-value significance levels can be used in this weighing procedure.

Finally, the posterior probability of weighted probability of co-localizations, which in turn gives rise to the probabilistic observation of a given potential priors candidate gene with significant GWAS signals, is then given as:
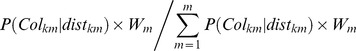



### Permutation for the significance of weighted probability of co-localizations

To adjust multiple testing, we used a permutation procedure to assess the significance of the significance level weighted probability of co-localizations, which then were converted to adjusted *p*-values. For each prior candidate gene, we first generated 1,000 randomized association sets each containing the same number of associations as the true data, sampled without replacement from all SNP loci on a chromosome. We then assigned each set of associations with the p-values (or RIMP counts, in this study) from the true associations and computed their posterior probability of co-localizations with each of the prior candidate genes. The lowest *p*-value from each permutation set was recorded as *Pe_j_^(random, i)^*  =  {*P_ej1_*, *P_ej2_*, …*P_ej1000_*}, for the *j*th prior candidate gene, the vector that serves as the distribution of null distribution of co-localization. The adjusted threshold was set to the 5% quantile of the *Pe_j_^(random, i)^*.

### Enrichment score calculation

In this study, we developed a statistical significance test for the observed enrichment, while preserving the structure of linkage disequilibrium in maize genome with linkage block and co-localization. For each prior candidate gene, the enrichment score *E_i_* is calculated by: 
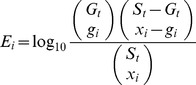
where *S_t_* is the total number of the SNP loci on a chromosome; *G_t_* is the total number of associations; *x_i_* is the number of SNP loci that are located in the linkage block of a prior candidate genes; and *g_i_* is the number of associations identified in the linkage block and also significantly co-locating with the target prior candidate gene.

Assuming every SNP locus on the chromosome has an equal probability to be in an association, we assessed the null hypothesis by randomizing association results with respect to SNP positions, while retaining the number of the associations. Enrichment scores are then compared with the null probability distribution of the target prior candidate genes. The cutoff threshold is preliminarily selected at LOD = 2.

### Gene ontology (GO) analysis

To further characterize the functionalities of maize flowering time a priori candidates, we conducted the GO analysis with a community tool, agriGO (www.bioinfo.cau.edu.cn/argiGO) that is built specifically for plant species [70]. All significantly enriched maize a priori candidates were input as the query list, and then maize genome loci were selected as the background references for the comparison. Both *p*-values converted from Z-scores and false discovery rates (FDR) for multiple tests correction are reported.

## Supporting Information

Figure S1 **Manhattan plot of maize days-to-silk GWAS associations.** The y-axis is in RMIP (re-sampling model inclusion probability) counts. Triangles pointing up are the QTLs that increase the days to silk flowering time in the comparison with B73, while triangles pointing down decrease the flowering time. Only significant associations showed.
(TIF)

Table S1 *Arabidopsis* flowering time related genes.
(PDF)

Table S2 Days-to-silk GWAS associations of maize NAM populations, RIMP≥2.
(PDF)

Table S3 Maize flowering time priori candidates and the annotation from their *Arabidopsis* homologs.
(PDF)

Table S4 GO analysis of maize flowering time priori candidate genes.
(PDF)

Table S5 *Arabidopsis* gene IDs for GO terms.
(PDF)
