# Supplementary figures and images for: PICARA, an Analytical Pipeline Providing Probabilistic Inference about A Priori Candidates Genes Underlying Genome-Wide Association QTL in Plants
Source: PLoS One. 2012 Nov 7;7(11):e46596. doi: 10.1371/journal.pone.0046596 (PMC3492367; doi:10.1371/journal.pone.0046596)

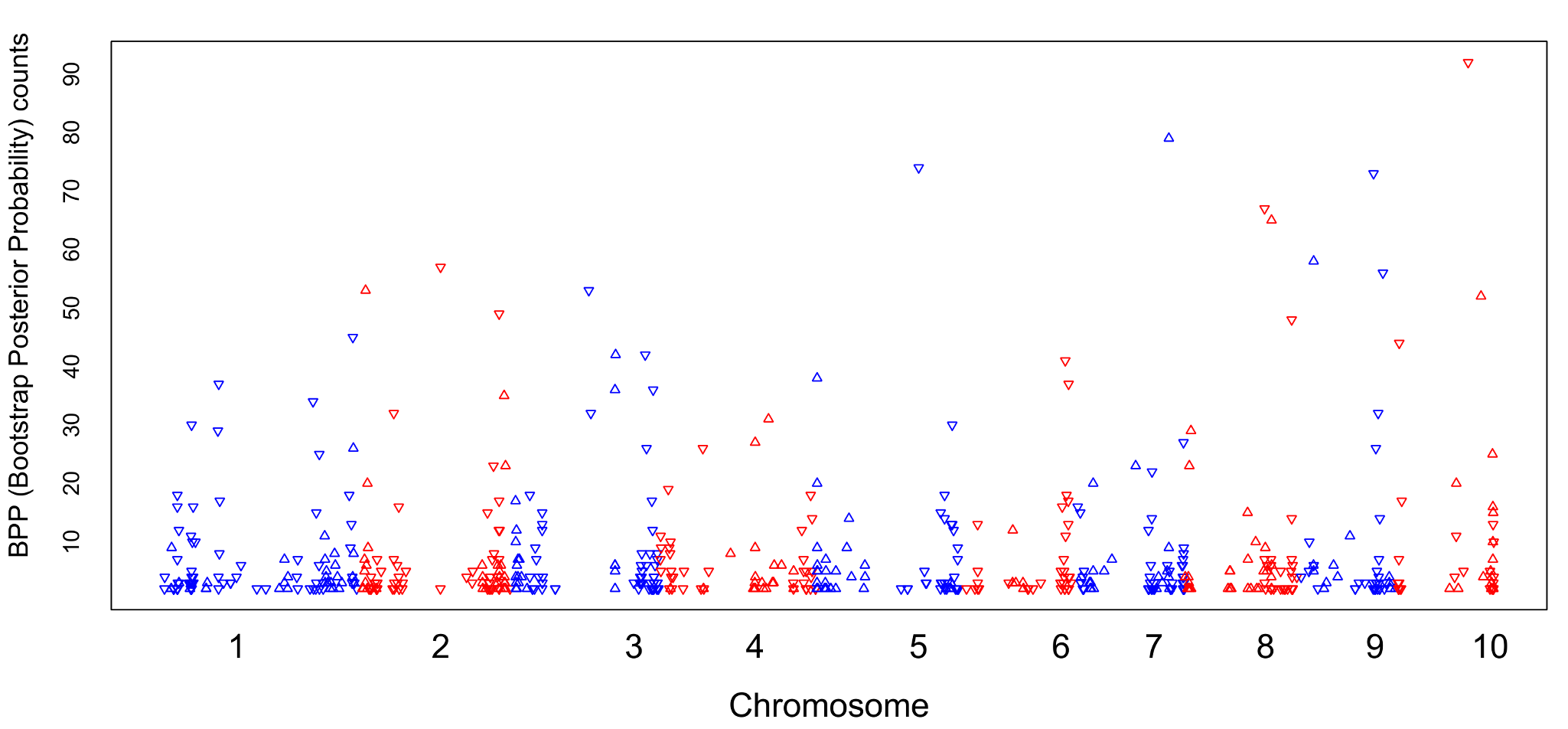

Supplement: Figure S1 — Manhattan plot of maize days-to-silk GWAS associations. The y-axis is in RMIP (re-sampling model inclusion probability) counts. Triangles pointing up are the QTLs that increase the days to silk flowering time in the comparison with B73, while triangles pointing down decrease the flowering time. Only significant associations showed. (TIF) [file pone.0046596.s001.tif]
