# Supplementary material for: PICARA, an Analytical Pipeline Providing Probabilistic Inference about A Priori Candidates Genes Underlying Genome-Wide Association QTL in Plants
Source: PLoS One. 2012 Nov 7;7(11):e46596. doi: 10.1371/journal.pone.0046596 (PMC3492367; doi:10.1371/journal.pone.0046596)
Supplement: Table S1 — Arabidopsis flowering time related genes. (PDF) [file pone.0046596.s002.pdf]

Table S1. *Arabidopsis* flowering time related genes

Locus id	chr	start	end	annotation	reference
AT1G01060	1	33379	37840	LHY	[1-3]
AT1G02340	1	465718	467844	HFR1	[4]
AT1G03970	1	1018100	1019247	GBF4	[2]
AT1G04400	1	1185549	1188516	CRY2	[1-3]
AT1G04440	1	1202254	1205802	CKL13	[1]
AT1G06040	1	1828412	1829889	STO	[1]
AT1G07980	1	2473165	2474927	NF-YC10	[5]
AT1G08620	1	2737031	2743724	PKDM7D	[6]
AT1G08970	1	2882543	2884337	NF-YC9	[5]
AT1G09030	1	2908611	2909030	NF-YB4	[5]
AT1G09530	1	3076584	3079541	PAP3; PIF3	[1,7]
AT1G09570	1	3095258	3100359	PHYA	[1-3]
AT1G09700	1	3137769	3140355	HYL1	[1]
AT1G10588	1	3501146	3501905	AT1G10588	[1]
AT1G12190	1	4394895	4396289	LWD1	[1,8]
AT1G12610	1	4289942	4291015	DDF1	[1,9]
AT1G12910	1	4394897	4396291	LWD1	[8]
AT1G13260	1	4542165	4543739	RAV1	[1,2]
AT1G14920	1	5149221	5151349	GAI	[1,10]
AT1G15550	1	5344473	5346161	GA4	[1]
AT1G17455	1	5997180	5998475	ELF4-L4	[2]
AT1G17590	1	6050222	6052541	NF-YA8	[5]
AT1G18100	1	6227217	6230188	MFT	[10]
AT1G18450	1	6348100	6351968	ATARP4	[1]
AT1G21970	1	7727577	7729617	NF-YB9	[5]
AT1G22690	1	8027287	8028114	AT1G22690	[1]
AT1G22770	1	8061833	8067705	GI	[1-3]
AT1G24260	1	8593631	8596087	SEPALLATA3	[1,11]
AT1G25540	1	8969052	8974647	PFT1	[1,2]
AT1G25560	1	8981677	8983041	TEM1	[2]
AT1G26310	1	9100140	9103590	CAL	[1,2]
AT1G26790	1	9273857	9275312	AtDOF1.3	[2]
AT1G29160	1	10183797	10184324	AtDOF1.5	[2]
AT1G30040	1	10537632	10539815	ATGA2OX2	[1]
AT1G30500	1	10804450	10806319	NF-YA7	[5]
AT1G30950	1	11036161	11037489	UFO	[1]
AT1G30960	1	11037593	11040014	AT1G30960	[1]
AT1G30970	1	11040262	11043732	SUF4	[1,2]
AT1G31814	1	11412589	11414483	FRL2	[1,10]
AT1G35160	1	12867181	12868793	GRF4	[2]
AT1G43700	1	16486671	16488681	VIP1	[1]
AT1G44090	1	16763117	16764926	ATGA20OX5	[1]
AT1G44446	1	16850799	16853664	CH1	[1]
AT1G45249	1	17165127	17167866	ABF2	[2]
AT1G47250	1	17319177	17321074	PAF2	[2]
AT1G48270	1	17831621	17834088	GCR1	[1]
AT1G49480	1	18317846	18320313	RTV1	[1]

AT1G49720	1	18400092	18402628	ABF1	[2]
AT1G50370	1	18658647	18661974	FYPP3	[12]
AT1G50680	1	18777601	18778614	RAVL2	[2]
AT1G50960	1	18893217	18895387	ATGA2OX7	[1]
AT1G51120	1	18938091	18939149	RAVL3	[2]
AT1G52800	1	19667712	19669030	AT1G52800	[1]
AT1G53090	1	19787020	19790570	SPA4	[1-3]
AT1G53160	1	19810087	19811276	SPL4	[1,3]
AT1G54160	1	20217336	20219452	NF-YA5	[5]
AT1G54830	1	20451609	20452671	NF-YC3	[5]
AT1G55080	1	20556678	20557921	AT1G55080	[1]
AT1G56170	1	21024764	21025883	NF-YC2	[5]
AT1G57820	1	21417835	21421611	ORTH2	[1]
AT1G59940	1	22069282	22070638	ARR3	[1]
AT1G60980	1	22456238	22457805	ATGA20OX4	[1]
AT1G61040	1	22486872	22489634	VIP5	[1]
AT1G62750	1	23237099	23240112	ATSCO1	[1]
AT1G62830	1	23368155	23270867	LDL1	[1,13]
AT1G63030	1	23371072	23372075	DDF2	[1,9]
AT1G65480	1	24335091	24337597	FT	[1-3]
AT1G66350	1	24751858	24753706	RGL1	[1,10]
AT1G66410	1	24774217	24775900	CAM4	[14]
AT1G68050	1	25512339	25514552	FKF1	[1-3]
AT1G68840	1	25880327	25881736	TEM2	[2]
AT1G69120	1	25985993	25989976	AP1	[1-3]
AT1G69570	1	26161528	26163496	AtDOF1.10	[2,15]
AT1G69935	1	26345464	26346789	AT1G69935	[1]
AT1G70170	1	26427537	26429019	MMP	[1]
AT1G71692	1	26956307	26958789	AGL12	[1]
AT1G72050	1	27118686	27121132	AT1G72050	[1]
AT1G72630	1	27344270	27345576	ELF4-L2	[2]
AT1G72830	1	27409118	27411630	HAP2C; NF-YA3	[1,5]
AT1G74660	1	28051237	28051788	MIF1	[1]
AT1G74670	1	28056947	28057810	AT1G74670	[1]
AT1G76710	1	28794623	28797570	ASHH1; SDG26	[1,16]
AT1G77080	1	28960531	28964990	FLM; MAF1	[1,17]
AT1G77300	1	29044816	29053704	EFS	[1,2]
AT1G78300	1	29461671	29463385	GRF2	[2]
AT1G78440	1	29516492	29517944	ATGA2OX1	[1]
AT1G79280	1	29824069	29837871	NUA	[1]
AT1G79460	1	29895285	29899480	GA2	[1]
AT1G79730	1	30005431	30008898	ELF7	[1,18]
AT1G80330	1	30202953	30204429	ATGA3OX4	[1]
AT1G80340	1	30205585	30207092	GA4H	[1]
AT1G80680	1	30328900	30333661	MOS3	[1]
AT2G01570	2	255248	257549	RGA1	[1,10]
AT2G02560	2	689787	697595	CAND1	[1]
AT2G02950	2	854946	856537	PKS1	[1]
AT2G03710	2	1129265	1131835	SEPALLATA4	[1]

AT2G04030	2	1281838	1286101	CR88	[1]
AT2G06210	2	2428900	2436684	ELF8	[1,18]
AT2G06255	2	2457573	2459811	ELF4-L3	[2]
AT2G13540	2	5636884	5642957	ABH1	[19]
AT2G13570	2	5655842	5656489	NF-YB7	[5]
AT2G14900	2	6411257	6412412	AT2G14900	[1]
AT2G17770	2	7723103	7724161	FDP	[2]
AT2G18040	2	7842164	7843870	PIN1AT	[20]
AT2G18790	2	8146963	8151512	PHYB	[1-3]
AT2G18870	2	8167471	8168668	VEL3	[2]
AT2G18880	2	8172189	8178995	VEL2	[2]
AT2G18915	2	8201654	8204656	LKP2	[1,2]
AT2G19520	2	8455936	8459525	FVE	[2]
AT2G20180	2	8711105	8713973	PIL5	[1]
AT2G21070	2	9047944	9050609	FIONA1	[1,21]
AT2G22540	2	9586954	9590973	SVP	[1-3]
AT2G22630	2	9625542	9629037	AGL17	[1,22]
AT2G23070	2	9823965	9826898	CK2alphaCp	[2]
AT2G23080	2	9827113	9829537	CK2A /CK2alphaC	[2]
AT2G23380	2	99626650	9967439	CLF	[1,2]
AT2G24790	2	10573977	10575224	COL3	[1,23]
AT2G25930	2	11066113	11070402	ELF3	[1,2]
AT2G26710	2	11387570	11390690	BAS1	[1]
AT2G27470	2	11744981	11746411	NF-YB11	[5]
AT2G27550	2	11780328	11781758	ATC	[1,10]
AT2G28190	2	12014498	12016569	CZS	[24]
AT2G28290	2	12063290	12080160	SYD	[1]
AT2G28550	2	12233028	12235620	TOE1	[1,2]
AT2G29950	2	12767585	12768435	ELF4-L1	[2]
AT2G30120	2	12860541	12861910	FLX	[25]
AT2G30810	2	13134903	13135743	AT2G30810	[1]
AT2G31650	2	13455272	13462181	ATX1	[26]
AT2G32950	2	13985010	13990612	COP1	[1-3]
AT2G33810	2	14312077	14313148	SPL3	[1,3]
AT2G33835	2	14318863	14321776	FES1	[1][[10]
AT2G34140	2	14413758	14414765	AtDOF2.3	[2]
AT2G34555	2	14564067	14565776	ATGA2OX3	[1]
AT2G34720	2	14649767	14651627	NF-YA4	[5]
AT2G34880	2	14711880	14716634	PKDM7C	[6]
AT2G35670	2	14992565	14996756	FIE2	[2]
AT2G36270	2	15204764	15207528	ABI5	[2]
AT2G37060	2	15576063	15577877	NF-YB8	[5]
AT2G37678	2	15808543	15809871	FHY1	[1]
AT2G38880	2	16238492	16240476	NF-YB1	[5]
AT2G39250	2	16395964	16398151	SNZ	[1,2]
AT2G39540	2	16507944	16508319	AT2G39540	[1]
AT2G39810	2	16619878	16625135	HOS1	[1,10]
AT2G40080	2	16741372	16741990	ELF4	[1,2]
AT2G41070	2	17130788	17132370	EEL	[2]

AT2G42200	2	17587407	17589630	SPL9	[3]
AT2G42590	2	17731855	17733967	GRF9	[27]
AT2G42830	2	17827332	17831090	SHP2	[1]
AT2G43010	2	17893504	17896127	PIF4	[1,28]
AT2G43410	2	18032324	18038320	FPA	[1,2]
AT2G44680	2	18433621	18435432	CKB4	[1,29]
AT2G45650	2	18811424	18813596	AGL6	[1,11,30,31]
AT2G45660	2	18814612	18818121	SOC1	[1-3]
AT2G46340	2	19029246	19034486	SPA1	[1-3]
AT2G46790	2	19239718	19242156	APRR9	[1,2]
AT2G46830	2	19252741	19255983	CCA1	[1,2]
AT2G47310	2	19430754	19434347	AT2G47310	[1]
AT2G47700	2	19559384	19561654	RF12	[1,32]
AT2G47810	2	19582750	19583618	NF-YB5	[5]
AT3G01460	3	173323	182461	MBD9	[1]
AT3G02310	3	464286	467081	SEPALLATA2	[1]
AT3G02380	3	487243	488700	COL2	[1,2]
AT3G02520	3	526437	528313	GRF7	[2]
AT3G02885	3	638028	639062	GASA5	[1]
AT3G03090	3	700463	704776	ATVGT1	[1]
AT3G03450	3	819337	821406	RGL2	[10]
AT3G04510	3	1215642	1216964	AT3G04510	[1]
AT3G04610	3	1250559	1254879	FLK	[1,2]
AT3G05040	3	1401277	1408203	HST	[1]
AT3G05120	3	1430477	1432784	ATGID1A; GID1/GAr2	[1,10,33,34]
AT3G05690	3	1676552	1678938	ATHAP2B; NF-YA2	[1,5]
AT3G06910	3	2178636	2181203	AT3G06910	[1]
AT3G06930	3	2185149	2189393	AtPRMT4b	[1]
AT3G07650	3	2441663	2444538	COL9	[1,35]
AT3G10185	3	3145584	3146204	AT3G10185	[1]
AT3G10390	3	3229298	3231824	FLD	[1,2]
AT3G11440	3	3602099	3605110	ATMYB65	[1]
AT3G11540	3	3631887	3637955	SPY	[2]
AT3G12480	3	3957777	3960472	NF-YC11	[5]
AT3G12810	3	4065049	4074085	PIE1	[1,2]
AT3G13550	3	4423349	4424883	FUS9	[36]
AT3G13682	3	4479200	4481516	LDL2	[1,13]
AT3G14020	3	4642968	4644301	NF-YA6	[5]
AT3G15270	3	5140372	5141355	SPL5	[1,3]
AT3G15354	3	5169102	5172844	SPA3	[1-3]
AT3G18990	3	6548875	6551859	VRN1	[1,2]
AT3G19040	3	6567163	6575288	HAF2	[1]
AT3G19140	3	6614910	6615335	DNF	[37]
AT3G19290	3	6687140	6690123	ABF4	[2]
AT3G19980	3	6961742	6965114	ATFYPP3	[1]
AT3G20550	3	7174470	7177948	DDL	[1]
AT3G20740	3	7248815	7252458	FIE	[1,2]
AT3G20910	3	7326382	7328576	NF-YA9	[5]
AT3G21320	3	7499059	7501847	putative EEC	[1,2]

AT3G22380	3	7912912	7919517	TIC	[1,38]
AT3G22590	3	8003934	8005579	PHP; CDC73	[39]
AT3G24440	3	8876034	8878178	VRN5	[1,2]
AT3G25730	3	9396420	9397747	ARF14	[2]
AT3G26120	3	9547635	9550423	TEL1	[1]
AT3G26640	3	9794457	9795694	LWD2	[1,8]
AT3G26790	3	9855065	9857226	FUS3	[1]
AT3G30180	3	11813216	11816244	BR6OX2	[1]
AT3G30260	3	11909119	11912880	AGL79	[2]
AT3G33520	3	14104642	14105635	ATARP6	[1,2]
AT3G44460	3	16079976	16081906	AtbZIP67	[2]
AT3G46640	3	17194075	17196203	PCL1; LUX	[1,2]
AT3G47500	3	17514985	17517043	CDF3	[1-3]
AT3G48430	3	17946594	17951731	REF6	[1,2]
AT3G48590	3	18008657	18009982	NF-YC1	[5]
AT3G50000	3	18534459	18536970	CKA2 / CK2alphaB	[2]
AT3G53340	3	19774331	19776205	NF-YB10	[5]
AT3G54220	3	20070158	20072780	SCR	[40]
AT3G54720	3	20265703	20268826	AMP1	[1]
AT3G54990	3	20384695	20387499	SMZ	[1,2]
AT3G56850	3	21046341	21048516	AREB3	[2]
AT3G57230	3	21188689	21191911	AGL16	[1]
AT3G57300	3	21210467	21218864	AT3G57300	[1]
AT3G57390	3	21244678	21236888	AGL18	[1,2]
AT3G58070	3	21517590	21518631	GIS	[1]
AT3G58780	3	21749437	21752884	SHP1	[1,41]
AT3G59060	3	21838955	21841484	PIL6	[1,4]
AT3G60250	3	22281312	22283088	CKB3	[1,42]
AT3G61120	3	22629234	22631466	AGL13	[1]
AT3G62090	3	22999522	23001684	PIL2; PIF6	[1,4]
AT3G63010	3	23300400	23302461	ATGID1B; GID1L2/GAr3	[1,10,33,34]
AT4G00450	4	203471	211003	CRP	[1]
AT4G00650	4	269026	271503	FRI	[1-3]
AT4G00690	4	281645	283129	AT4G00690	[1]
AT4G01060	4	460472	461085	ETC3	[1]
AT4G02020	4	886600	891955	EZA1; SWN1	[1,43]
AT4G02560	4	1123490	1128421	LD	[1,2]
AT4G02780	4	1237767	1244813	GA1	[1,10]
AT4G03400	4	1497535	1499864	DFL2	[1]
AT4G04885	4	2471972	2475981	PCFS4	[44]
AT4G04890	4	2476487	2482343	PDF2	[1]
AT4G05420	4	2746121	2752943	DDB1A	[45]
AT4G08920	4	5724100	5727250	CRY1	[1-3]
AT4G09000	4	5775210	5777481	GRF1	[2]
AT4G09610	4	6074767	6075642	GASA2	[1]
AT4G09960	4	6236473	6240681	STK	[41]
AT4G10180	4	6346463	6349276	DET1	[45,46]
AT4G11110	4	6771601	6777221	SPA2	[1-3]
AT4G11880	4	7143109	7147216	AGL14	[1,2]

AT4G12480	4	7406102	7406934	pEARLI1	[1]
AT4G14110	4	8132881	8134915	COP9	[1]
AT4G14540	4	8344616	8345218	NF-YB3	[5]
AT4G14690	4	8418278	8419258	ELIP2	[1]
AT4G15090	4	8614063	8618142	FAR1	[1,47]
AT4G15180	4	8651406	8662587	AT4G15180	[1]
AT4G15880	4	9012660	9016131	ESD4	[1,10]
AT4G16250	4	9195617	9199501	PHYD	[1,2]
AT4G16280	4	9206613	9214841	FCA	[1,2]
AT4G16780	4	9449133	9450762	HAT4	[1]
AT4G16810	4	9459889	9462272	B3H7I2	[1,2]
AT4G16845	4	9476162	9479897	VRN2	[1,2]
AT4G17640	4	9825210	9827285	CKB2	[1,42]
AT4G18130	4	10042149	10046094	PHYE	[1,2]
AT4G18960	4	10382856	10388539	AG	[48,49]
AT4G20370	4	11000782	11003007	TSF	[1-3]
AT4G20400	4	11008666	11013842	PKDM7B	[6]
AT4G21100	4	11258761	11265463	DDB1B	[45]
AT4G21200	4	11302695	11306611	ATGA2OX8	[1]
AT4G21690	4	11527241	11529072	ATGA3OX3	[1]
AT4G22140	4	11727738	11730521	EBS	[1,10]
AT4G22950	4	12023926	12027432	AGL19	[1,2]
AT4G23340	4	12195463	12196803	AT4G23340	[1]
AT4G24210	4	12563553	12564482	SLY1	[10]
AT4G24520	4	12990894	12992468	GA5	[1]
AT4G24540	4	12670975	12674082	AGL24	[1-3]
AT4G24620	4	12708762	12712835	PGI1	[1]
AT4G25530	4	13038369	13042452	FWA	[1,13]
AT4G26000	4	13197246	13199836	PEP	[2]
AT4G27060	4	13581407	13585161	TOR1	[1]
AT4G27430	4	13718685	13723330	CIP7	[1]
AT4G29130	4	14352043	14355109	ATHXK1	[1]
AT4G29830	4	14597667	14599306	VIP3	[1,50]
AT4G30200	4	14786639	14790509	VEL1	[1,2]
AT4G31120	4	15132017	15136645	ATPRMT5; SKB1	[1,51]
AT4G31160	4	15145853	15153075	DCAF1	[45]
AT4G31380	4	15229791	15230724	FLP1	[1]
AT4G31500	4	15273477	15275316	CYP83B1	[1]
AT4G32040	4	15494071	15493262	KNAT5	[1]
AT4G32980	4	15914725	15918047	ATH1	[1,52]
AT4G33280	4	16047358	16049359	AT4G33280	[1]
AT4G34000	4	16295558	16298255	ABF3	[2]
AT4G34530	4	16498391	16500174	CIB1	[2]
AT4G35900	4	17004598	17006290	FD	[1-3]
AT4G36920	4	17400844	17403329	AP2	[1,2]
AT4G37580	4	17658606	17660872	HLS1	[1]
AT4G37940	4	17835689	17838615	AGL21	[1,11]
AT4G39400	4	18324655	18328820	BRI1	[1]
AT5G01400	5	162550	171072	ESP4	[53]

AT5G02200	5	437458	438892	FHL	[1]
AT5G02810	5	637895	641975	PRR7	[1,54]
AT5G02840	5	648702	651970	LCL1	[1]
AT5G03790	5	1004982	1006372	ATHB51	[1]
AT5G03840	5	1024640	1025811	TFL1	[1,2]
AT5G04240	5	1169545	1174879	ELF6	[1,2]
AT5G05690	5	1702689	1706788	CPD	[1]
AT5G06100	5	1837908	1840728	MYB33	[1,10]
AT5G06510	5	1984991	1987044	NF-YA10	[5]
AT5G07200	5	2243554	2245340	YAP169	[1]
AT5G08190	5	2635902	2637433	NF-YB12	[5]
AT5G08330	5	2680745	2681814	CHE	[1,2]
AT5G09230	5	2871474	2873779	SIR2; SRT2	[2,55]
AT5G10140	5	3173498	3179449	FLC	[1-3]
AT5G10450	5	3283853	3286317	GRF6	[2]
AT5G10625	5	3358788	3359782	AT5G10625	[1]
AT5G11260	5	3593381	3594993	HY5	[1]
AT5G11530	5	3696863	3701549	EMF1	[1]
AT5G12840	5	4050694	4053609	HAP2A; NF-YA1	[1,5]
AT5G13480	5	4326531	4331702	FY	[1,2]
AT5G13790	5	4449017	4450846	AGL15	[1,11]
AT5G14920	5	4826482	4827983	AT5G14920	[1]
AT5G15230	5	4944903	4946219	GASA4	[1]
AT5G15800	5	5151334	5154154	SEP_1	[11,30]
AT5G15840	5	5171185	5172761	CO	[1,3,10]
AT5G15850	5	5176094	5177900	COL1	[1,2]
AT5G15960	5	5209901	5210730	KIN1	[1]
AT5G15970	5	5211914	5212668	KIN2	[1]
AT5G16050	5	5243745	5245820	GRF5	[2]
AT5G16260	5	5311207	5315767	ELF9	[2]
AT5G16320	5	5344505	5346022	FRL1	[1,10]
AT5G17690	5	5827173	5829684	TFL2	[1,10,56]
AT5G19550	5	6598019	6601821	ASP2	[1]
AT5G20570	5	6956660	6958311	RBX1	[32]
AT5G20730	5	7016447	7022115	NPH4	[1]
AT5G23090	5	7749070	7750544	NF-YB13	[5]
AT5G23150	5	7785838	7792492	HUA2	[1,2]
AT5G23260	5	7836096	7838505	AGL32	[2]
AT5G24470	5	8355954	8358876	APRR5	[1,2]
AT5G24860	5	8541781	8542452	FPF1	[1,10]
AT5G24930	5	8589234	8591236	AtCOL4	[2]
AT5G25810	5	8986774	8987790	TNY	[1]
AT5G25900	5	9036021	9038409	GA3	[1]
AT5G27220	5	9578757	9582752	A_TM021B04.7	[2]
AT5G27230	5	9584095	9588052	AT5G27230	[1]
AT5G27320	5	9629090	9631213	ATGID1C; GID1L3/GAr1	[1,10,33,34]
AT5G27910	5	9940736	9941299	NF-YC8	[5]
AT5G28450	5	10372942	10374194	AT5G28450	[1]
AT5G28490	5	10454397	10455200	LSH1	[1]

AT5G35840	5	14025056	14028994	PHYC	[1,2]
AT5G37055	5	14641551	14642440	SEF1	[57]
AT5G37770	5	15016084	15016849	TCH2	[1]
AT5G38140	5	15220208	15222272	NF-YC12	[5]
AT5G38150	5	15240346	15242177	PMI15	[1]
AT5G38480	5	15409697	15411516	GRF3	[2]
AT5G39660	5	15895927	15898272	CDF2	[1-3]
AT5G41360	5	16561568	16566508	XPB2	[1]
AT5G41790	5	16744758	16750075	CIP1	[1]
AT5G41990	5	16794967	16798006	WNK8	[58]
AT5G42400	5	16954469	16960671	SDG25	[59]
AT5G42790	5	17159044	17161070	PAF1	[2]
AT5G42910	5	17203742	17205380	AtbZIP15	[2]
AT5G43250	5	17356174	17356566	NF-YC13	[5]
AT5G44080	5	17738652	17739873	bzip13	[2]
AT5G46210	5	18748645	18754037	CUL4	[1,60]
AT5G46910	5	19065007	19068107	AT5G46910	[1]
AT5G47010	5	19089236	19096561	LBA1	[1]
AT5G47080	5	19141839	19143838	CKB1	[1,42]
AT5G47640	5	19309227	19310272	NF-YB2	[5]
AT5G47670	5	19315023	19316169	NF-YB6	[5]
AT5G48150	5	19539481	19541924	PAT1	[1]
AT5G49020	5	19888477	19892146	AtPRMT4a	[1]
AT5G49230	5	19958873	19960544	HRB1	[61]
AT5G50470	5	20555120	20555758	NF-YC7	[5]
AT5G50480	5	20557574	20558487	NF-YC6	[5]
AT5G50490	5	20560434	20561187	NF-YC5	[5]
AT5G51230	5	20840962	20846790	EMF2	[1,2]
AT5G51310	5	20870080	20871944	AT5G51310	[1]
AT5G51810	5	21072414	21074034	AT2353	[1]
AT5G51820	5	21080594	21085283	PGM	[1]
AT5G53360	5	21647902	21649567	SINAT5	[62,63]
AT5G54270	5	22038273	22039568	CAB1A; LHCB3	[64]
AT5G54510	5	22148319	22150904	DFL1	[1]
AT5G55390	5	22447966	22454414	EDM2	[65]
AT5G57360	5	23258653	23261816	ZTL	[1,2]
AT5G57380	5	23263621	23266730	VIN3	[1,2]
AT5G57660	5	23355464	23356989	ATCOL5	[2]
AT5G58230	5	23573238	23575471	MSI1	[1,2]
AT5G59560	5	24017782	24019183	SRR1	[1,66]
AT5G59570	5	24021114	24022738	AT5G59570	[1]
AT5G59710	5	24074633	24079144	VIP2	[1]
AT5G59845	5	24128550	24129246	AT5G59845	[1]
AT5G60100	5	24215225	24218590	APRR3	[1,2]
AT5G60120	5	24225012	24228950	TOE2	[1,2]
AT5G60910	5	24519708	24523369	AGL8; FUL	[1,2,67]
AT5G61150	5	24620882	24624951	VIP4	[1,68]
AT5G61270	5	24638775	24640600	PIF7	[4]
AT5G61380	5	24692290	24695776	TOC1	[1,2]

AT5G61850	5	24861521	24864159	LFY	[1,2]
AT5G62040	5	24940036	24940935	BFT	[1,2]
AT5G62430	5	25086319	25088160	CDF1	[1-3]
AT5G62640	5	25166659	25169767	ELF5	[1,10]
AT5G63310	5	25371904	25373861	NDPK2	[69]
AT5G63470	5	25415701	25417098	NF-YC4	[5]
AT5G64813	5	25927505	25930122	LIP1	[1]
AT5G65050	5	25999480	26003552	AGL31; MAF2	[1,2]
AT5G65060	5	26004655	26008541	MAF3	[1,2]
AT5G65070	5	26009486	26013360	MAF4	[1,2]
AT5G65080	5	26014730	26019691	AGL68	[1,2]
AT5G65430	5	26148201	26150342	GRF8	[2]
AT5G65540	5	26212915	26215548	AT5G65540	[1]
AT5G67100	5	26794220	26802330	ICU2	[1]
AT5G67180	5	26801949	26804249	TOE3	[2]
AT5G67380	5	26880966	26883430	CKA1	[2]

References

1. Brachi B, Faure N, Horton M, Flahauw E, Vazquez A, et al. (2010) Linkage and association mapping of Arabidopsis thaliana flowering time in nature. PLoS Genet 6: e1000940.
2. Higgins JA, Bailey PC, Laurie DA (2010) Comparative genomics of flowering time pathways using Brachypodium distachyon as a model for the temperate grasses. PLoS One 5: e10065.
3. Amasino R (2010) Seasonal and developmental timing of flowering. Plant J 61: 1001-1013.
4. Yamashino T, Matsushika A, Fujimori T, Sato S, Kato T, et al. (2003) A Link between circadian-controlled bHLH factors and the APRR1/TOC1 quintet in Arabidopsis thaliana. Plant Cell Physiol 44: 619-629.
5. Kumimoto RW, Zhang Y, Siefers N, Holt BF, 3rd (2010) NF-YC3, NF-YC4 and NF-YC9 are required for CONSTANS-mediated, photoperiod-dependent flowering in Arabidopsis thaliana. Plant J.
6. Yang W, Jiang D, Jiang J, He Y (2010) A plant-specific histone H3 lysine 4 demethylase represses the floral transition in Arabidopsis. Plant J 62: 663-673.
7. Ni M, Tepperman JM, Quail PH (1998) PIF3, a phytochrome-interacting factor necessary for normal photoinduced signal transduction, is a novel basic helix-loop-helix protein. Cell 95: 657-667.
8. Wu JF, Wang Y, Wu SH (2008) Two new clock proteins, LWD1 and LWD2, regulate Arabidopsis photoperiodic flowering. Plant Physiol 148: 948-959.
9. Magome H, Yamaguchi S, Hanada A, Kamiya Y, Oda K (2004) dwarf and delayed-flowering 1, a novel Arabidopsis mutant deficient in gibberellin biosynthesis because of overexpression of a putative AP2 transcription factor. Plant J 37: 720-729.
10. Flowers JM, Hanzawa Y, Hall MC, Moore RC, Purugganan MD (2009) Population genomics of the Arabidopsis thaliana flowering time gene network. Mol Biol Evol 26: 2475-2486.
11. Gomez-Mena C, de Folter S, Costa MM, Angenent GC, Sablowski R (2005) Transcriptional program controlled by the floral homeotic gene AGAMOUS during early organogenesis. Development 132: 429-438.
12. Kim DH, Kang JG, Yang SS, Chung KS, Song PS, et al. (2002) A phytochrome-associated protein phosphatase 2A modulates light signals in flowering time control in Arabidopsis. Plant Cell 14: 3043-3056.
13. Jiang D, Yang W, He Y, Amasino RM (2007) Arabidopsis relatives of the human lysine-specific Demethylase1 repress the expression of FWA and FLOWERING LOCUS C and thus promote the floral transition. Plant Cell 19: 2975-2987.
14. McCormack E, Tsai YC, Braam J (2005) Handling calcium signaling: Arabidopsis CaMs and CMLs. Trends Plant Sci 10: 383-389.
15. Fornara F, Panigrahi KC, Gissot L, Sauerbrunn N, Ruhl M, et al. (2009) Arabidopsis DOF transcription factors act redundantly to reduce CONSTANS

expression and are essential for a photoperiodic flowering response. Dev Cell 17: 75-86.
16. Xu L, Zhao Z, Dong A, Soubigou-Taconnat L, Renou JP, et al. (2008) Di- and tri- but not monomethylation on histone H3 lysine 36 marks active transcription of genes involved in flowering time regulation and other processes in Arabidopsis thaliana. Mol Cell Biol 28: 1348-1360.
17. Scortecci KC, Michaels SD, Amasino RM (2001) Identification of a MADS-box gene, FLOWERING LOCUS M, that represses flowering. Plant J 26: 229-236.
18. He Y, Doyle MR, Amasino RM (2004) PAF1-complex-mediated histone methylation of FLOWERING LOCUS C chromatin is required for the vernalization-responsive, winter-annual habit in Arabidopsis. Genes Dev 18: 2774-2784.
19. Bezerra IC, Michaels SD, Schomburg FM, Amasino RM (2004) Lesions in the mRNA cap-binding gene ABA HYPERSENSITIVE 1 suppress FRIGIDA-mediated delayed flowering in Arabidopsis. Plant J 40: 112-119.
20. Wang Y, Liu C, Yang D, Yu H, Liou YC (2010) Pin1At encoding a peptidyl-prolyl cis/trans isomerase regulates flowering time in Arabidopsis. Mol Cell 37: 112-122.
21. Kim J, Kim Y, Yeom M, Kim JH, Nam HG (2008) FIONA1 is essential for regulating period length in the Arabidopsis circadian clock. Plant Cell 20: 307-319.
22. Han P, Garcia-Ponce B, Fonseca-Salazar G, Alvarez-Buylla ER, Yu H (2008) AGAMOUS-LIKE 17, a novel flowering promoter, acts in a FT-independent photoperiod pathway. Plant J 55: 253-265.
23. Datta S, Hettiarachchi GH, Deng XW, Holm M (2006) Arabidopsis CONSTANS-LIKE3 is a positive regulator of red light signaling and root growth. Plant Cell 18: 70-84.
24. Krichevsky A, Kozlovsky SV, Tian GW, Chen MH, Zaltsman A, et al. (2007) How pollen tubes grow. Dev Biol 303: 405-420.
25. Andersson CR, Helliwell CA, Bagnall DJ, Hughes TP, Finnegan EJ, et al. (2008) The FLX gene of Arabidopsis is required for FRI-dependent activation of FLC expression. Plant Cell Physiol 49: 191-200.
26. Pien S, Fleury D, Mylne JS, Crevillen P, Inze D, et al. (2008) ARABIDOPSIS TRITHORAX1 dynamically regulates FLOWERING LOCUS C activation via histone 3 lysine 4 trimethylation. Plant Cell 20: 580-588.
27. Mayfield JD, Folta KM, Paul AL, Ferl RJ (2007) The 14-3-3 Proteins mu and upsilon influence transition to flowering and early phytochrome response. Plant Physiol 145: 1692-1702.
28. Brock MT, Maloof JN, Weinig C (2010) Genes underlying quantitative variation in ecologically important traits: PIF4 (phytochrome interacting factor 4) is associated with variation in internode length, flowering time, and fruit set in Arabidopsis thaliana. Molecular Ecology 19: 1187-1199.
29. Portoles S, Mas P (2007) Altered oscillator function affects clock resonance and is responsible for the reduced day-length sensitivity of CKB4 overexpressing plants. Plant J 51: 966-977.

30. Ma H, Yanofsky MF, Meyerowitz EM (1991) AGL1-AGL6, an Arabidopsis gene family with similarity to floral homeotic and transcription factor genes. Genes Dev 5: 484-495.
31. Koo SC, Bracko O, Park MS, Schwab R, Chun HJ, et al. (2010) Control of lateral organ development and flowering time by the Arabidopsis thaliana MADS-box Gene AGAMOUS-LIKE6. Plant J 62: 807-816.
32. Chen M, Ni M (2006) RFI2, a RING-domain zinc finger protein, negatively regulates CONSTANS expression and photoperiodic flowering. Plant J 46: 823-833.
33. Griffiths J, Murase K, Rieu I, Zentella R, Zhang ZL, et al. (2006) Genetic characterization and functional analysis of the GID1 gibberellin receptors in Arabidopsis. Plant Cell 18: 3399-3414.
34. Iuchi S, Suzuki H, Kim YC, Iuchi A, Kuromori T, et al. (2007) Multiple loss-of-function of Arabidopsis gibberellin receptor AtGID1s completely shuts down a gibberellin signal. Plant J 50: 958-966.
35. Cheng XF, Wang ZY (2005) Overexpression of COL9, a CONSTANS-LIKE gene, delays flowering by reducing expression of CO and FT in Arabidopsis thaliana. Plant J 43: 758-768.
36. Wei N, Kwok SF, von Arnim AG, Lee A, McNellis TW, et al. (1994) Arabidopsis COP8, COP10, and COP11 genes are involved in repression of photomorphogenic development in darkness. Plant Cell 6: 629-643.
37. Morris K, Thornber S, Codrai L, Richardson C, Craig A, et al. (2010) DAY NEUTRAL FLOWERING represses CONSTANS to prevent Arabidopsis flowering early in short days. Plant Cell 22: 1118-1128.
38. Ding Z, Millar AJ, Davis AM, Davis SJ (2007) TIME FOR COFFEE encodes a nuclear regulator in the Arabidopsis thaliana circadian clock. Plant Cell 19: 1522-1536.
39. Yu X, Michaels SD (2010) The Arabidopsis Paf1c complex component CDC73 participates in the modification of FLOWERING LOCUS C chromatin. Plant Physiol 153: 1074-1084.
40. Cui H, Benfey PN (2009) Interplay between SCARECROW, GA and LIKE HETEROCHROMATIN PROTEIN 1 in ground tissue patterning in the Arabidopsis root. Plant J 58: 1016-1027.
41. Losa A, Colombo M, Brambilla V, Colombo L (2010) Genetic interaction between AINTEGUMENTA (ANT) and the ovule identity genes SEEDSTICK (STK), SHATTERPROOF1 (SHP1) and SHATTERPROOF2 (SHP2). Sex Plant Reprod 23: 115-121.
42. Sugano S, Andronis C, Green RM, Wang ZY, Tobin EM (1998) Protein kinase CK2 interacts with and phosphorylates the Arabidopsis circadian clock-associated 1 protein. Proc Natl Acad Sci U S A 95: 11020-11025.
43. Chanvivattana Y, Bishopp A, Schubert D, Stock C, Moon YH, et al. (2004) Interaction of Polycomb-group proteins controlling flowering in Arabidopsis. Development 131: 5263-5276.
44. Xing D, Zhao H, Xu R, Li QQ (2008) Arabidopsis PCFS4, a homologue of yeast polyadenylation factor Pcf11p, regulates FCA alternative processing and promotes flowering time. Plant J 54: 899-910.

45. Zhang Y, Feng S, Chen F, Chen H, Wang J, et al. (2008) Arabidopsis DDB1-CUL4 ASSOCIATED FACTOR1 forms a nuclear E3 ubiquitin ligase with DDB1 and CUL4 that is involved in multiple plant developmental processes. Plant Cell 20: 1437-1455.
46. Chen H, Huang X, Gusmaroli G, Terzaghi W, Lau OS, et al. (2010) Arabidopsis CULLIN4-damaged DNA binding protein 1 interacts with CONSTITUTIVELY PHOTOMORPHOGENIC1-SUPPRESSOR OF PHYA complexes to regulate photomorphogenesis and flowering time. Plant Cell 22: 108-123.
47. Lin R, Wang H (2004) Arabidopsis FHY3/FAR1 gene family and distinct roles of its members in light control of Arabidopsis development. Plant Physiol 136: 4010-4022.
48. Moyroud E, Minguet EG, Ott F, Yant L, Pose D, et al. (2011) Prediction of Regulatory Interactions from Genome Sequences Using a Biophysical Model for the Arabidopsis LEAFY Transcription Factor. Plant Cell 23: 1293-1306.
49. Mizukami Y, Ma H (1997) Determination of Arabidopsis floral meristem identity by AGAMOUS. Plant Cell 9: 393-408.
50. Zhang H, Ransom C, Ludwig P, van Nocker S (2003) Genetic analysis of early flowering mutants in Arabidopsis defines a class of pleiotropic developmental regulator required for expression of the flowering-time switch flowering locus C. Genetics 164: 347-358.
51. Wang X, Zhang Y, Ma Q, Zhang Z, Xue Y, et al. (2007) SKB1-mediated symmetric dimethylation of histone H4R3 controls flowering time in Arabidopsis. EMBO J 26: 1934-1941.
52. Proveniers M, Rutjens B, Brand M, Smeekens S (2007) The Arabidopsis TALE homeobox gene ATH1 controls floral competency through positive regulation of FLC. Plant J 52: 899-913.
53. Herr AJ, Molnar A, Jones A, Baulcombe DC (2006) Defective RNA processing enhances RNA silencing and influences flowering of Arabidopsis. Proc Natl Acad Sci U S A 103: 14994-15001.
54. Nakamichi N, Kita M, Niinuma K, Ito S, Yamashino T, et al. (2007) Arabidopsis clock-associated pseudo-response regulators PRR9, PRR7 and PRR5 coordinately and positively regulate flowering time through the canonical CONSTANS-dependent photoperiodic pathway. Plant Cell Physiol 48: 822-832.
55. Bond DM, Wilson IW, Dennis ES, Pogson BJ, Jean Finnegan E (2009) VERNALIZATION INSENSITIVE 3 (VIN3) is required for the response of Arabidopsis thaliana seedlings exposed to low oxygen conditions. Plant J 59: 576-587.
56. Mylne JS, Barrett L, Tessadori F, Mesnage S, Johnson L, et al. (2006) LHP1, the Arabidopsis homologue of HETEROCHROMATIN PROTEIN1, is required for epigenetic silencing of FLC. Proc Natl Acad Sci U S A 103: 5012-5017.
57. Choi K, Park C, Lee J, Oh M, Noh B, et al. (2007) Arabidopsis homologs of components of the SWR1 complex regulate flowering and plant development. Development 134: 1931-1941.

58. Wang Y, Liu K, Liao H, Zhuang C, Ma H, et al. (2008) The plant WNK gene family and regulation of flowering time in Arabidopsis. Plant Biol (Stuttg) 10: 548-562.
59. Berr A, Xu L, Gao J, Cognat V, Steinmetz A, et al. (2009) SET DOMAIN GROUP25 encodes a histone methyltransferase and is involved in FLOWERING LOCUS C activation and repression of flowering. Plant Physiol 151: 1476-1485.
60. Bernhardt A, Lechner E, Hano P, Schade V, Dieterle M, et al. (2006) CUL4 associates with DDB1 and DET1 and its downregulation affects diverse aspects of development in Arabidopsis thaliana. Plant J 47: 591-603.
61. Kang X, Zhou Y, Sun X, Ni M (2007) HYPERSENSITIVE TO RED AND BLUE 1 and its C-terminal regulatory function control FLOWERING LOCUS T expression. Plant J 52: 937-948.
62. Park BS, Sang WG, Yeu SY, Choi YD, Paek N-C, et al. (2007) Post-translational regulation of FLC is mediated by an E3 ubiquitin ligase activity of SINAT5 in Arabidopsis. Plant Science 173: 269-275.
63. Park BS, Eo HJ, Jang IC, Kang HG, Song JT, et al. (2010) Ubiquitination of LHY by SINAT5 regulates flowering time and is inhibited by DET1. Biochem Biophys Res Commun 398: 242-246.
64. Damkjaer JT, Kereiche S, Johnson MP, Kovacs L, Kiss AZ, et al. (2009) The photosystem II light-harvesting protein Lhcb3 affects the macrostructure of photosystem II and the rate of state transitions in Arabidopsis. Plant Cell 21: 3245-3256.
65. Eulgem T, Tsuchiya T, Wang XJ, Beasley B, Cuzick A, et al. (2007) EDM2 is required for RPP7-dependent disease resistance in Arabidopsis and affects RPP7 transcript levels. Plant J 49: 829-839.
66. Staiger D, Allenbach L, Salathia N, Fiechter V, Davis SJ, et al. (2003) The Arabidopsis SRR1 gene mediates phyB signaling and is required for normal circadian clock function. Genes Dev 17: 256-268.
67. Teper-Bamnolker P, Samach A (2005) The flowering integrator FT regulates SEPALLATA3 and FRUITFULL accumulation in Arabidopsis leaves. Plant Cell 17: 2661-2675.
68. Zhang H, van Nocker S (2002) The VERNALIZATION INDEPENDENCE 4 gene encodes a novel regulator of FLOWERING LOCUS C. Plant J 31: 663-673.
69. Schmitz RJ, Sung S, Amasino RM (2008) Histone arginine methylation is required for vernalization-induced epigenetic silencing of FLC in winter-annual Arabidopsis thaliana. Proc Natl Acad Sci U S A 105: 411-416.
