# Supplementary material for: PICARA, an Analytical Pipeline Providing Probabilistic Inference about A Priori Candidates Genes Underlying Genome-Wide Association QTL in Plants
Source: PLoS One. 2012 Nov 7;7(11):e46596. doi: 10.1371/journal.pone.0046596 (PMC3492367; doi:10.1371/journal.pone.0046596)
Supplement: Table S2 — Days-to-silk GWAS associations of maize NAM populations, RIMP≥2. (PDF) [file pone.0046596.s003.pdf]

Table S2. Days-to-silk GWAS associations of maize NAM populations, RIMP≥ 2.

SNP.ID	CHR	Position	RIMP score	Effect	Alleles	pvalue
PZE0102343672	1	2343672	2	0.298443216	C/A	2.46E-07
PZE0102739639	1	2739639	4	0.177198958	G/T	4.97E-07
PZE0112441177	1	12441177	2	-0.245073815	CG/--	1.31E-07
PZE0113053270	1	13053270	9	-0.289918056	A/C	2.74E-07
PZE0116071333	1	16071333	2	0.285890103	---/AGC	4.47E-07
PZE0116081021	1	16081021	2	0.284614418	T/C	8.30E-08
PZE0116399802	1	16399802	2	0.277866934	C/A	2.31E-07
PZE0121391422	1	21391422	2	0.30881962	C/T	1.10E-11
PZE0121691747	1	21691747	16	0.230784575	C/T	4.91E-08
PZE0121691748	1	21691748	7	0.250519451	T/C	3.45E-10
PZE0121692012	1	21692012	18	0.271175602	T/C	2.20E-08
PZE0122035462	1	22035462	2	0.230358896	C/G	1.86E-08
PZE0122035583	1	22035583	2	0.223624601	G/C	1.39E-08
PZE0122465559	1	22465559	3	0.220928295	CA/--	2.65E-07
PZE0123806620	1	23806620	3	0.361546432	G/A	6.64E-10
PZE0123989134	1	23989134	12	0.30187281	C/A	1.31E-07
PZE0124145984	1	24145984	3	-0.421349609	C/T	1.98E-07
PZE0138937153	1	38937153	3	0.293639811	T/C	1.22E-08
PZE0140591431	1	40591431	3	0.296089512	A/G	1.30E-10
PZE0140610904	1	40610904	2	-0.423809543	G/C	7.08E-08
PZE0141762101	1	41762101	2	0.425575344	G/T	1.13E-08
PZE0142176060	1	42176060	11	0.534129784	-------/GTGGAGG	2.65E-07
PZE0142678765	1	42678765	3	0.528854566	C/T	8.23E-09
PZE0142682449	1	42682449	2	0.369052375	A/G	2.63E-09
PZE0142970281	1	42970281	5	0.311093653	C/T	1.31E-07
PZE0143013025	1	43013025	2	0.315362384	A/C	8.22E-17
PZE0143030453	1	43030453	2	0.207315574	C/G	5.59E-07
PZE0143030456	1	43030456	4	0.292708275	G/A	1.85E-13
PZE0143030620	1	43030620	30	0.315067152	TAGA/----	1.38E-08
PZE0144027363	1	44027363	3	0.231266946	A/G	6.96E-09
PZE0144192914	1	44192914	10	0.232228386	A/G	1.17E-06
PZE0145309466	1	45309466	4	0.226597474	A/C	9.70E-13
PZE0145438322	1	45438322	16	0.327045307	T/G	7.84E-08
PZE0145438461	1	45438461	3	0.319529866	T/G	1.69E-07
PZE0145673038	1	45673038	3	0.329102659	T/C	4.03E-06
PZE0148812426	1	48812426	10	0.219227034	T/-	1.50E-06
PZE0164829467	1	64829467	2	-0.248650155	T/-	1.20E-07
PZE0164910609	1	64910609	2	-0.250773484	G/A	1.18E-07
PZE0166618479	1	66618479	3	-0.425706995	C/A	5.50E-08
PZE0182574078	1	82574078	29	0.323475408	G/C	1.37E-11
PZE0183455543	1	83455543	4	0.346842481	G/C	1.09E-23
PZE0183521852	1	83521852	2	0.278398123	T/C	1.30E-11
PZE0183796358	1	83796358	37	0.354557464	G/A	1.75E-07
PZE0184764219	1	84764219	8	0.350853659	T/C	3.12E-26
PZE0185375888	1	85375888	17	0.370615991	A/G	2.73E-12
PZE0190910978	1	90910978	3	0.282951285	C/A	1.45E-07

PZE01101421889	1	101421889	3	0.274752386	A/T	5.88E-08
PZE01101783898	1	101783898	3	0.280546754	A/G	1.26E-08
PZE01110462763	1	110462763	4	0.342900719	G/A	3.65E-07
PZE01117222419	1	117222419	6	0.396072306	C/T	1.68E-07
PZE01141017792	1	141017792	2	0.421877295	G/A	2.22E-08
PZE01154545974	1	154545974	2	0.242832323	-/A	2.24E-07
PZE01174754436	1	174754436	2	-0.412638634	C/G	5.74E-10
PZE01182470745	1	182470745	7	-0.388903503	T/C	2.19E-07
PZE01182480696	1	182480696	3	-0.227175373	C/G	2.51E-07
PZE01187246215	1	187246215	2	-0.224684872	G/A	2.10E-07
PZE01187743913	1	187743913	4	-0.220135703	A/C	1.83E-07
PZE01201024687	1	201024687	4	0.293898853	C/A	3.56E-07
PZE01202454730	1	202454730	7	0.47261202	G/A	2.81E-07
PZE01202454741	1	202454741	2	0.358296007	C/G	1.01E-07
PZE01220839213	1	220839213	2	0.388846047	T/C	7.55E-08
PZE01225365825	1	225365825	34	0.310694552	G/A	1.07E-09
PZE01226125241	1	226125241	2	0.331495577	C/T	3.20E-12
PZE01229526841	1	229526841	2	0.485639583	A/G	9.77E-12
PZE01230247159	1	230247159	3	0.432532107	T/C	3.37E-11
PZE01230248196	1	230248196	15	0.647902921	GCTTCCA/-------	5.16E-10
PZE01234376245	1	234376245	6	0.694138945	C/T	2.59E-13
PZE01234783216	1	234783216	25	0.641576193	------/GCGGCG	8.84E-09
PZE01234903720	1	234903720	2	0.24075091	T/C	3.20E-08
PZE01240151043	1	240151043	3	0.315496458	A/-	1.66E-07
PZE01240585818	1	240585818	2	0.301555007	C/T	0.00031269
PZE01242870282	1	242870282	11	-0.376680127	C/T	1.06E-07
PZE01243394093	1	243394093	7	-0.290201667	G/A	2.33E-07
PZE01243425772	1	243425772	3	-0.310912525	--/CG	3.26E-08
PZE01244830581	1	244830581	4	-0.436995274	CATG/----	7.70E-08
PZE01245518780	1	245518780	5	-0.30468348	C/A	5.19E-08
PZE01247517986	1	247517986	3	-0.316455327	A/G	1.58E-07
PZE01251183151	1	251183151	2	-0.409571527	T/A	8.30E-10
PZE01252291207	1	252291207	3	-0.366046281	C/T	4.43E-08
PZE01255347223	1	255347223	2	-0.478930812	G/A	1.58E-07
PZE01257733428	1	257733428	8	-0.262128492	C/G	2.79E-08
PZE01258826896	1	258826896	6	-0.371324146	C/A	9.90E-08
PZE01262053573	1	262053573	4	-0.328864957	T/A	1.73E-08
PZE01264521564	1	264521564	2	-0.220318919	C/A	1.10E-09
PZE01268545915	1	268545915	3	-0.288667985	G/A	8.88E-08
PZE01279723445	1	279723445	18	0.498411528	T/C	4.54E-08
PZE01281483945	1	281483945	9	0.387826919	A/G	9.82E-08
PZE01282959397	1	282959397	13	0.359126199	A/T	3.20E-09
PZE01284150628	1	284150628	3	0.443358862	A/T	1.22E-11
PZE01284311932	1	284311932	4	0.580516968	---/CGT	2.77E-10
PZE01284936299	1	284936299	4	-0.252207477	A/G	9.03E-09
PZE01285155499	1	285155499	45	0.430969343	G/C	1.72E-06
PZE01285908241	1	285908241	26	-0.283116253	-------/GCTGCAG	1.31E-07
PZE01286226511	1	286226511	8	-0.325840502	C/T	1.41E-07
PZE01286864289	1	286864289	3	-0.325505954	T/G	4.05E-08

PZE01287311493	1	287311493	3	0.448008483	---/GTA	6.54E-07
PZE01290826051	1	290826051	2	0.390112043	G/C	3.74E-10
PZE0208761036	2	8761036	2	-0.339426756	G/A	3.18E-07
PZE0209330964	2	9330964	2	-0.22257759	-/A	2.10E-07
PZE0209969167	2	9969167	2	-0.235239299	G/A	2.91E-07
PZE0211850186	2	11850186	7	-0.366805543	----/TTGT	6.03E-07
PZE0211852413	2	11852413	5	-0.282767607	T/A	2.10E-06
PZE0213200943	2	13200943	3	-0.254753063	A/G	1.81E-08
PZE0213437067	2	13437067	53	-0.343872523	G/A	1.75E-07
PZE0214637518	2	14637518	6	-0.339505545	A/G	4.13E-09
PZE0216045218	2	16045218	2	-0.274679887	A/C	2.22E-06
PZE0216214626	2	16214626	20	-0.362150468	T/-	9.11E-10
PZE0217112733	2	17112733	9	-0.41208009	C/T	1.05E-08
PZE0218514116	2	18514116	2	0.468069081	G/A	2.49E-07
PZE0220449395	2	20449395	2	0.321432367	----/ACAA	1.11E-09
PZE0221546927	2	21546927	4	0.425996022	-/G	7.15E-08
PZE0221549158	2	21549158	2	0.267463778	T/C	5.78E-08
PZE0221646110	2	21646110	2	0.336460578	T/G	3.62E-10
PZE0221653557	2	21653557	2	0.25027725	G/A	1.64E-08
PZE0222903059	2	22903059	2	-0.187076857	--/CG	3.76E-07
PZE0223635496	2	23635496	2	-0.474011026	G/A	8.10E-12
PZE0227513830	2	27513830	3	0.434024319	----/TCCG	9.30E-08
PZE0229453359	2	29453359	2	-0.232047619	C/G	2.50E-07
PZE0229694116	2	29694116	2	0.333978475	A/G	2.57E-10
PZE0229838349	2	29838349	2	0.24977441	G/T	4.52E-09
PZE0230667229	2	30667229	2	0.264858546	C/G	8.87E-08
PZE0230667336	2	30667336	7	0.228782906	G/T	1.18E-08
PZE0230730080	2	30730080	3	0.276460275	C/A	4.03E-13
PZE0230737519	2	30737519	2	0.243086409	G/A	1.36E-10
PZE0230999462	2	30999462	3	0.297836209	G/A	2.64E-07
PZE0236803984	2	36803984	5	0.248632996	C/T	5.99E-08
PZE0254056862	2	54056862	2	0.317366963	A/C	1.27E-12
PZE0255750434	2	55750434	32	0.273096043	---/GGC	4.54E-09
PZE0255750715	2	55750715	7	0.284436309	G/A	1.60E-08
PZE0256511993	2	56511993	2	0.406970027	T/C	4.21E-09
PZE0256512019	2	56512019	3	0.25750635	C/T	2.77E-12
PZE0260766697	2	60766697	6	0.263887429	C/T	4.89E-10
PZE0261165755	2	61165755	3	0.232593237	T/C	2.91E-09
PZE0263250796	2	63250796	16	0.329829006	C/G	2.25E-05
PZE0263250819	2	63250819	4	0.344493938	----/ATCA	1.95E-16
PZE0263828507	2	63828507	2	0.537770898	--/GC	1.18E-10
PZE0263835561	2	63835561	2	0.289034291	T/A	3.78E-09
PZE0268516120	2	68516120	2	0.257352465	C/G	6.87E-12
PZE0269097444	2	69097444	3	0.324008945	A/G	1.37E-10
PZE0273992510	2	73992510	5	0.606999365	G/A	2.13E-10
PZE02125795197	2	125795197	2	0.344749826	-/C	1.85E-11
PZE02126020893	2	126020893	57	0.395936099	C/T	1.25E-08
PZE02164975847	2	164975847	4	0.563437618	G/A	8.92E-08
PZE02173487874	2	173487874	3	0.578671155	AC/--	1.08E-07

PZE02174283885	2	174283885	5	0.605314254	G/A	2.14E-08
PZE02188442924	2	188442924	6	-0.311509673	A/T	3.67E-07
PZE02188982985	2	188982985	3	-0.528128868	A/G	2.89E-07
PZE02189202213	2	189202213	4	-0.315636062	C/G	9.57E-08
PZE02189436852	2	189436852	2	-0.40639721	G/A	4.81E-07
PZE02194107669	2	194107669	2	-0.293249179	A/G	3.88E-07
PZE02196446699	2	196446699	15	0.272407402	G/A	3.86E-08
PZE02196447103	2	196447103	2	0.360820318	GTTGCG/------	3.46E-08
PZE02199062470	2	199062470	2	0.305387028	C/G	4.55E-07
PZE02199256842	2	199256842	3	0.256606142	G/A	4.33E-08
PZE02199256911	2	199256911	3	0.196543859	C/T	1.44E-07
PZE02200712385	2	200712385	4	0.342605266	----/ATCG	4.65E-08
PZE02201337414	2	201337414	2	0.266525155	A/G	2.11E-14
PZE02203612407	2	203612407	7	0.288509861	C/T	5.35E-09
PZE02203613248	2	203613248	2	0.281879175	T/C	1.81E-07
PZE02205448177	2	205448177	23	0.231414923	G/A	2.46E-08
PZE02206169457	2	206169457	8	0.274028243	A/C	5.70E-08
PZE02211045102	2	211045102	4	0.307991665	A/C	2.47E-07
PZE02211562587	2	211562587	3	-0.290844052	G/A	3.66E-07
PZE02212021538	2	212021538	6	-0.422037523	---/CAT	1.76E-06
PZE02212960866	2	212960866	2	-0.43550018	-/A	8.28E-08
PZE02213510665	2	213510665	12	0.397492923	A/C	8.72E-31
PZE02213909345	2	213909345	2	0.382733586	G/A	2.16E-31
PZE02213913155	2	213913155	17	0.35226237	T/G	7.07E-20
PZE02213913279	2	213913279	7	0.347226498	T/C	4.68E-22
PZE02213913485	2	213913485	49	0.347310897	A/G	1.11E-08
PZE02213917446	2	213917446	2	0.290437284	-----/TCTGA	9.19E-08
PZE02214339260	2	214339260	5	-0.363927367	C/T	2.32E-08
PZE02214423590	2	214423590	4	-0.700098942	C/A	4.02E-07
PZE02214674047	2	214674047	12	0.35116635	G/C	6.32E-10
PZE02215991830	2	215991830	2	-0.619699265	C/G	2.59E-08
PZE02216525566	2	216525566	6	-0.404390169	G/-	1.27E-07
PZE02220852484	2	220852484	2	-0.303712174	C/G	1.71E-07
PZE02221439621	2	221439621	35	-0.347046647	G/C	1.00E-07
PZE02221890429	2	221890429	3	-0.4301035	C/T	2.09E-07
PZE02222054415	2	222054415	4	-0.398667037	G/T	5.38E-08
PZE02222694241	2	222694241	2	-0.438206099	T/C	6.19E-09
PZE02222782014	2	222782014	3	-0.377633524	G/A	2.86E-07
PZE02223532702	2	223532702	23	-0.264504586	C/T	1.24E-07
PZE02223589777	2	223589777	2	-0.27131533	G/A	2.84E-09
PZE02230250417	2	230250417	2	0.468612053	G/A	3.86E-07
PZE0308341413	3	8341413	17	-0.364191819	C/G	1.81E-08
PZE0308610971	3	8610971	10	-0.362244648	T/C	2.25E-08
PZE0308618579	3	8618579	4	-0.183708173	T/-	8.77E-09
PZE0308906285	3	8906285	2	-0.25158723	--/GG	1.05E-08
PZE0309113211	3	9113211	2	-0.239326636	T/A	4.77E-09
PZE0309227204	3	9227204	3	-0.247017642	G/A	6.33E-09
PZE0309676416	3	9676416	12	-0.235164556	G/A	4.34E-08
PZE0310013038	3	10013038	2	-0.222969808	A/G	1.09E-09

PZE0310386485	3	10386485	6	-0.244948621	--/TA	2.26E-08
PZE0311925031	3	11925031	4	-0.339043139	T/C	8.46E-09
PZE0311927247	3	11927247	7	-0.229162964	A/T	3.98E-10
PZE0312966038	3	12966038	3	-0.23775873	G/T	1.38E-12
PZE0314162249	3	14162249	7	-0.282617559	T/C	1.18E-08
PZE0317305493	3	17305493	4	-0.380239861	G/C	3.78E-10
PZE0325653380	3	25653380	2	-0.341851259	------/AAAAGT	2.53E-09
PZE0329727316	3	29727316	18	0.307288457	C/T	6.23E-11
PZE0329763057	3	29763057	4	0.284533176	C/T	7.20E-10
PZE0333776289	3	33776289	2	0.301475559	C/T	4.93E-17
PZE0333776490	3	33776490	2	0.285619022	G/A	2.92E-13
PZE0336758850	3	36758850	2	0.243469461	G/A	1.61E-10
PZE0337924339	3	37924339	9	0.415826863	G/A	7.26E-08
PZE0339485775	3	39485775	7	0.766852269	C/T	1.08E-06
PZE0343008281	3	43008281	4	0.281796464	A/T	4.75E-10
PZE0348293519	3	48293519	2	0.329531643	T/A	1.78E-13
PZE0348307218	3	48307218	15	0.263616799	A/G	2.23E-11
PZE0348308090	3	48308090	12	0.278781521	C/T	8.29E-11
PZE0348342221	3	48342221	13	0.314222466	TG/--	3.92E-11
PZE0348579555	3	48579555	3	0.2224918	A/G	5.59E-12
PZE0349162852	3	49162852	4	0.464619354	---/AGG	5.07E-06
PZE0367958527	3	67958527	2	0.278251074	G/A	1.63E-09
PZE0367991775	3	67991775	2	0.242391174	T/G	2.63E-08
PZE03118293736	3	118293736	53	0.590181287	AGC/---	3.21E-09
PZE03121387143	3	121387143	32	0.550364525	----/GCGT	8.77E-06
PZE03157651220	3	157651220	6	-0.355636099	A/G	6.37E-19
PZE03157651234	3	157651234	36	-0.367742666	C/T	3.32E-15
PZE03157651412	3	157651412	6	-0.364365372	----/CGGG	3.02E-19
PZE03157725327	3	157725327	2	-0.445610396	C/T	7.03E-25
PZE03157752813	3	157752813	5	-0.429417791	------/GGCGGA	5.47E-22
PZE03158538927	3	158538927	42	-0.485517192	T/A	3.10E-08
PZE03185790280	3	185790280	3	0.236902698	A/G	6.21E-08
PZE03193118425	3	193118425	2	0.218669865	G/A	2.14E-08
PZE03194891814	3	194891814	3	0.231588808	G/A	3.75E-07
PZE03195595219	3	195595219	4	0.190775719	C/G	3.51E-08
PZE03195843691	3	195843691	3	0.221462454	T/C	2.55E-07
PZE03196575213	3	196575213	6	0.227094268	-----/TAACA	1.08E-07
PZE03197295000	3	197295000	3	0.221431053	G/T	9.47E-08
PZE03197461182	3	197461182	3	0.226358672	C/-	6.82E-09
PZE03197461398	3	197461398	3	0.249744528	C/T	3.79E-08
PZE03197461431	3	197461431	4	0.238148497	G/C	1.46E-07
PZE03197861394	3	197861394	8	0.248270955	C/A	6.83E-10
PZE03198005603	3	198005603	5	0.235668831	T/C	2.04E-08
PZE03198609559	3	198609559	3	0.234282184	C/T	9.69E-08
PZE03203005602	3	203005602	42	0.245156115	A/C	0.000183378
PZE03204791389	3	204791389	6	0.182802449	T/C	9.50E-06
PZE03204949298	3	204949298	26	0.193138966	T/G	0.00087511
PZE03208721571	3	208721571	2	0.363800239	C/A	1.46E-07
PZE03210055424	3	210055424	8	0.2405048	T/C	1.06E-05

PZE03210989573	3	210989573	3	0.272398998	T/C	1.32E-16
PZE03212241075	3	212241075	2	0.269380013	C/T	4.02E-19
PZE03212942868	3	212942868	17	0.267591136	A/G	3.25E-12
PZE03213854276	3	213854276	2	0.231649831	C/T	7.50E-10
PZE03214085717	3	214085717	12	0.239926889	A/C	3.79E-13
PZE03214172163	3	214172163	2	0.278287797	A/C	4.39E-14
PZE03214679049	3	214679049	2	0.409692131	AAG/---	9.65E-08
PZE03214897638	3	214897638	36	0.226059358	C/T	1.68E-09
PZE03215885662	3	215885662	3	0.319338756	T/C	1.30E-12
PZE03215885959	3	215885959	4	0.359684991	G/C	8.75E-08
PZE03215993354	3	215993354	2	0.207142792	C/A	8.13E-12
PZE03216042476	3	216042476	4	0.284133731	G/C	2.38E-09
PZE03216273891	3	216273891	3	0.484518254	A/G	1.49E-07
PZE03217720951	3	217720951	6	0.325422033	C/T	4.79E-08
PZE03220325438	3	220325438	2	0.368321808	G/C	7.25E-08
PZE03221072374	3	221072374	7	0.697516849	A/G	1.87E-07
PZE03221072587	3	221072587	8	0.408482764	G/A	8.08E-08
PZE03221216400	3	221216400	3	0.72391813	C/T	1.54E-07
PZE03221243468	3	221243468	3	0.478611519	-------/TCACCAT	1.07E-07
PZE03222010981	3	222010981	2	0.700209104	C/T	1.09E-07
PZE03222999303	3	222999303	2	0.645989903	G/A	6.73E-07
PZE0402852707	4	2852707	7	0.210942309	G/C	3.49E-07
PZE0403132779	4	3132779	11	0.194958745	T/C	2.26E-07
PZE0403546026	4	3546026	5	0.14926141	CAC/---	4.12E-07
PZE0403816990	4	3816990	9	0.18739406	A/T	1.75E-07
PZE0414596329	4	14596329	19	0.650167459	C/T	1.65E-11
PZE0416092562	4	16092562	9	0.66236311	A/C	2.63E-11
PZE0416175289	4	16175289	2	0.375245687	A/G	5.09E-07
PZE0417307125	4	17307125	8	0.634689869	T/C	1.29E-09
PZE0417963013	4	17963013	10	0.417502037	C/T	2.59E-07
PZE0417968231	4	17968231	2	-0.382425344	A/T	1.94E-07
PZE0418975560	4	18975560	4	0.618395218	G/T	2.76E-09
PZE0418975984	4	18975984	5	0.600682193	G/A	6.58E-08
PZE0420050438	4	20050438	2	0.541338469	C/T	7.04E-09
PZE0421768743	4	21768743	4	0.343102327	A/G	1.80E-07
PZE0436854121	4	36854121	2	0.534526306	C/G	3.50E-07
PZE0438073634	4	38073634	5	0.982493823	A/G	6.34E-15
PZE0463838959	4	63838959	2	0.343846817	C/T	6.50E-07
PZE0466515756	4	66515756	26	0.540920789	T/C	7.72E-09
PZE0467508024	4	67508024	2	-0.379886515	C/T	5.90E-08
PZE0467935455	4	67935455	2	0.825630281	G/A	2.69E-19
PZE0475293318	4	75293318	5	0.792580676	A/C	1.96E-15
PZE04108097627	4	108097627	8	-0.419191643	G/T	1.56E-07
PZE04142187936	4	142187936	2	-0.341989529	T/A	3.54E-11
PZE04143390615	4	143390615	2	-0.22748084	C/G	3.96E-07
PZE04144474828	4	144474828	3	-0.436825131	G/A	3.38E-08
PZE04145005984	4	145005984	9	-0.407625699	C/T	1.29E-08
PZE04145058158	4	145058158	27	-0.390319028	G/A	6.32E-10
PZE04145252892	4	145252892	2	-0.21890085	C/G	4.93E-07

PZE04145253219	4	145253219	4	-0.249189088	C/G	1.58E-11
PZE04155328023	4	155328023	3	-0.342326145	G/A	1.16E-09
PZE04157426945	4	157426945	2	-0.222465028	C/T	5.37E-10
PZE04161571880	4	161571880	2	-0.241754644	C/G	5.60E-11
PZE04165062843	4	165062843	31	-0.224539303	G/T	5.05E-09
PZE04170875008	4	170875008	3	-0.385970466	C/T	9.86E-09
PZE04172644792	4	172644792	3	-0.239665781	-------/CCAGACC	1.49E-07
PZE04173636300	4	173636300	6	-0.308547841	G/A	2.05E-07
PZE04184636742	4	184636742	6	-0.384943461	C/G	2.91E-07
PZE04202199289	4	202199289	3	0.295884247	C/T	2.92E-08
PZE04202554358	4	202554358	2	0.415625728	G/T	6.34E-09
PZE04202773454	4	202773454	5	-0.459335306	AA/--	3.79E-07
PZE04202774666	4	202774666	2	-0.319070192	-----/CACCC	3.06E-07
PZE04215074267	4	215074267	12	0.228999389	A/G	1.36E-09
PZE04217654480	4	217654480	7	0.537139829	C/T	1.46E-08
PZE04217859383	4	217859383	5	0.244400954	T/C	3.64E-09
PZE04217868119	4	217868119	3	0.221797593	C/A	3.53E-07
PZE04221605444	4	221605444	2	0.79669005	C/T	6.12E-09
PZE04221605684	4	221605684	5	0.225618166	T/G	2.97E-12
PZE04221669007	4	221669007	2	0.332654082	C/T	9.15E-08
PZE04221758441	4	221758441	2	0.751238301	T/A	8.95E-08
PZE04221842627	4	221842627	2	0.796076949	-------/ACATAGA	4.32E-10
PZE04228107499	4	228107499	2	0.21910334	G/A	3.03E-08
PZE04228366544	4	228366544	18	0.549651029	T/G	8.07E-09
PZE04229071102	4	229071102	4	0.259129794	A/G	7.40E-09
PZE04230533018	4	230533018	14	0.295487189	---/GGA	4.26E-09
PZE04230533187	4	230533187	5	0.22917137	G/C	8.61E-08
PZE04235568964	4	235568964	2	0.209326395	G/C	3.51E-08
PZE04235779639	4	235779639	2	0.236585341	------/GAAAAA	4.79E-10
PZE0501851072	5	1851072	2	-0.217370556	A/G	1.47E-10
PZE0502141423	5	2141423	3	-0.198750716	T/C	1.15E-09
PZE0502273106	5	2273106	38	-0.59852556	C/T	1.14E-09
PZE0502320362	5	2320362	20	-0.375355826	C/A	3.45E-10
PZE0502321454	5	2321454	5	-0.20241731	TGATC/-----	3.93E-10
PZE0502333715	5	2333715	2	-0.379642793	G/C	2.86E-09
PZE0502333776	5	2333776	9	-0.330697327	T/C	8.38E-08
PZE0502583875	5	2583875	6	-0.202630686	A/G	1.37E-07
PZE0502767798	5	2767798	5	-0.311583948	-------/GGCCTCC	9.35E-10
PZE0503379699	5	3379699	2	-0.225451624	G/C	2.97E-09
PZE0513318830	5	13318830	2	-0.31858399	CC/--	5.55E-08
PZE0514550290	5	14550290	2	-0.323736715	A/T	4.67E-08
PZE0514550552	5	14550552	3	-0.423938291	G/A	1.37E-07
PZE0515335585	5	15335585	7	-0.41504109	C/T	6.59E-08
PZE0515440613	5	15440613	5	-0.285681287	T/A	1.08E-07
PZE0515979938	5	15979938	2	-0.302156768	T/-	2.02E-07
PZE0522632845	5	22632845	2	-0.488582536	C/T	4.55E-08
PZE0522676420	5	22676420	2	-0.346316009	A/T	5.10E-08
PZE0530312238	5	30312238	2	-0.233710254	G/A	8.89E-08
PZE0530434945	5	30434945	5	-0.341039012	G/T	7.70E-08

PZE0546854470	5	46854470	9	-0.411223635	G/A	1.25E-07
PZE0550107289	5	50107289	14	-0.309773006	T/C	7.69E-08
PZE0553818311	5	53818311	4	-0.403041286	G/A	1.08E-07
PZE0572330761	5	72330761	2	-0.484642707	A/G	2.02E-07
PZE0573966374	5	73966374	6	-0.429281534	G/A	1.83E-08
PZE0574188372	5	74188372	4	-0.379239685	G/T	2.25E-07
PZE05128421645	5	128421645	2	0.214749672	A/G	4.08E-08
PZE05138077229	5	138077229	2	0.328859049	G/A	9.93E-12
PZE05138077238	5	138077238	2	0.316894091	C/G	6.36E-11
PZE05154772545	5	154772545	74	0.406059727	T/A	1.63E-09
PZE05163396564	5	163396564	5	0.353155969	T/C	4.31E-11
PZE05165529900	5	165529900	3	0.433084729	C/T	8.97E-11
PZE05166371871	5	166371871	3	0.348690868	C/G	2.65E-11
PZE05187542985	5	187542985	2	0.270397325	G/A	2.20E-08
PZE05187826004	5	187826004	15	0.409645449	------/CACTCG	6.79E-08
PZE05188218300	5	188218300	3	0.338297635	G/T	3.03E-08
PZE05189370001	5	189370001	3	0.229758582	G/T	8.12E-08
PZE05193590766	5	193590766	14	0.255307059	GA/--	1.82E-06
PZE05193590857	5	193590857	3	0.240542177	A/C	1.05E-07
PZE05193620808	5	193620808	18	0.364999794	A/G	4.62E-08
PZE05194099214	5	194099214	2	0.465738847	G/A	2.07E-07
PZE05195724382	5	195724382	2	-0.381847812	-/A	3.83E-08
PZE05196739226	5	196739226	2	0.450436708	C/A	3.99E-15
PZE05197785554	5	197785554	3	0.293138679	A/G	1.22E-08
PZE05200537084	5	200537084	2	-0.254562951	C/T	3.31E-07
PZE05202816707	5	202816707	2	-0.248789526	T/C	2.68E-08
PZE05202940068	5	202940068	3	-0.307277755	C/T	1.38E-07
PZE05202940182	5	202940182	3	-0.286071646	-/T	4.42E-07
PZE05203627184	5	203627184	13	0.352099842	A/T	9.82E-11
PZE05205072262	5	205072262	30	0.562042522	T/C	1.92E-08
PZE05205076398	5	205076398	13	0.624992242	T/-	3.13E-10
PZE05207106380	5	207106380	12	0.577733234	----/GATC	9.56E-09
PZE05208148943	5	208148943	4	0.482256226	C/T	1.24E-07
PZE05210062776	5	210062776	2	0.219850836	---/GCT	6.41E-07
PZE05212070039	5	212070039	2	0.175024937	C/G	5.77E-07
PZE05213281663	5	213281663	7	0.203058169	A/-	2.46E-07
PZE05213376649	5	213376649	9	0.161211905	C/T	1.52E-07
PZE05213471935	5	213471935	2	0.207574846	T/G	3.47E-07
PZE05214443752	5	214443752	2	0.151950809	A/G	4.86E-07
PZE0612073101	6	12073101	2	0.181882287	G/A	2.43E-07
PZE0624765445	6	24765445	2	0.207731544	A/C	2.53E-07
PZE0628774455	6	28774455	13	0.310625994	C/T	2.84E-07
PZE0628948098	6	28948098	2	0.199800835	C/T	1.15E-07
PZE0628974993	6	28974993	3	0.21956223	T/C	2.62E-07
PZE0628975046	6	28975046	5	0.216180851	G/A	7.67E-08
PZE0630659832	6	30659832	2	0.260013843	G/C	3.82E-09
PZE0675408195	6	75408195	3	0.286795189	A/G	1.52E-07
PZE0681211885	6	81211885	3	-0.226704772	G/A	5.02E-07
PZE0681547765	6	81547765	12	-0.272635459	G/A	2.54E-07

PZE0686163241	6	86163241	3	-0.305693112	C/A	4.81E-07
PZE0696872617	6	96872617	3	-0.256005449	G/T	1.21E-07
PZE0697537195	6	97537195	2	-0.411065811	---/CAC	8.65E-08
PZE06104104693	6	104104693	2	0.394960354	T/G	1.33E-07
PZE06104875703	6	104875703	2	0.290873241	A/C	1.20E-07
PZE06111892978	6	111892978	2	0.216834513	C/G	2.41E-08
PZE06123595644	6	123595644	3	0.340582666	G/C	8.50E-08
PZE06153390115	6	153390115	3	0.278926597	C/A	5.78E-08
PZE06153459652	6	153459652	3	0.528501531	-/T	1.63E-07
PZE06153807726	6	153807726	5	0.390700222	----/CATG	1.72E-07
PZE06153933354	6	153933354	2	0.23594985	G/C	6.06E-07
PZE06155799605	6	155799605	16	0.393966426	G/A	1.65E-07
PZE06157387216	6	157387216	3	0.201675587	G/A	7.20E-08
PZE06158017623	6	158017623	7	0.419279494	A/G	2.86E-07
PZE06158566790	6	158566790	5	0.469522112	-----/TCGGG	6.97E-08
PZE06158622875	6	158622875	4	0.226412462	C/T	1.13E-09
PZE06159350446	6	159350446	2	0.385937127	-/A	2.30E-07
PZE06159881934	6	159881934	2	0.233411733	T/C	3.87E-07
PZE06160357487	6	160357487	41	0.242014792	G/A	3.05E-09
PZE06160828364	6	160828364	11	0.340764426	T/C	4.19E-08
PZE06162221436	6	162221436	18	0.304467638	T/A	3.24E-09
PZE06164901038	6	164901038	17	0.253221052	C/T	5.11E-08
PZE06164902257	6	164902257	13	0.245043123	T/G	1.59E-09
PZE06165088726	6	165088726	2	0.251156798	C/A	2.39E-07
PZE06165111439	6	165111439	2	0.203216882	G/A	1.28E-07
PZE06165337617	6	165337617	37	0.261481243	-----/GCGTG	1.11E-07
PZE06165591160	6	165591160	2	0.233455988	G/C	3.75E-08
PZE06165668468	6	165668468	4	0.172191741	T/A	9.67E-09
PZE06166910015	6	166910015	4	0.289064784	G/A	2.34E-07
PZE06169047723	6	169047723	3	0.187883646	A/G	1.10E-08
PZE0709935471	7	9935471	16	0.229892827	A/G	5.23E-07
PZE0713866582	7	13866582	15	0.343929707	C/A	2.79E-07
PZE0714522242	7	14522242	3	0.225418992	G/C	1.91E-07
PZE0714522613	7	14522613	3	0.226637676	--/GG	3.06E-07
PZE0715217480	7	15217480	4	-0.415816284	C/G	9.08E-08
PZE0717583201	7	17583201	4	0.219688056	T/C	3.70E-08
PZE0717741690	7	17741690	2	0.606459859	G/A	4.54E-07
PZE0718575380	7	18575380	2	0.370000241	A/C	6.04E-09
PZE0718921530	7	18921530	2	0.229698021	A/G	7.06E-07
PZE0719332124	7	19332124	5	0.264621511	C/A	1.50E-07
PZE0727712736	7	27712736	3	-0.5352417	C/A	5.95E-08
PZE0728778060	7	28778060	2	-0.261806509	C/T	2.85E-07
PZE0729633259	7	29633259	3	-0.579219165	A/G	3.07E-09
PZE0730095623	7	30095623	2	-0.58934134	G/T	1.37E-09
PZE0733205423	7	33205423	20	-0.408792247	T/A	9.29E-09
PZE0733236905	7	33236905	5	-0.51770196	G/A	3.10E-09
PZE0734067606	7	34067606	2	-0.506320624	C/T	3.81E-09
PZE0749651392	7	49651392	5	-0.656420979	C/T	4.84E-12
PZE0761316039	7	61316039	7	-0.480006145	G/A	1.05E-07

PZE0796970855	7	96970855	23	-0.466371337	G/A	5.91E-08
PZE07116125798	7	116125798	2	0.21724722	G/A	6.52E-07
PZE07116156977	7	116156977	2	0.203514269	T/G	5.27E-08
PZE07116254055	7	116254055	12	0.325946255	AG/--	8.88E-11
PZE07117086311	7	117086311	2	0.299908894	G/A	2.83E-14
PZE07118139587	7	118139587	4	0.302099954	T/C	1.01E-10
PZE07118766596	7	118766596	3	0.240684152	G/C	1.23E-12
PZE07119745988	7	119745988	6	0.266830867	G/T	6.50E-10
PZE07120773076	7	120773076	2	0.505715486	A/C	2.99E-11
PZE07121162429	7	121162429	14	0.227948853	T/C	2.38E-11
PZE07121551354	7	121551354	22	0.27328008	G/A	2.61E-08
PZE07122142045	7	122142045	2	0.255501507	T/G	4.77E-08
PZE07124223655	7	124223655	2	0.253562209	GG/--	8.96E-08
PZE07124466064	7	124466064	3	0.247602242	C/G	3.69E-10
PZE07125260358	7	125260358	2	0.185087191	C/A	2.63E-08
PZE07128347669	7	128347669	2	-0.369938454	G/C	1.93E-07
PZE07131352556	7	131352556	4	-0.288946945	G/A	3.59E-07
PZE07136947759	7	136947759	3	-0.348178721	G/A	2.15E-07
PZE07144421018	7	144421018	5	-0.326320034	-/G	3.88E-16
PZE07144428241	7	144428241	6	0.285910577	C/T	1.02E-06
PZE07146526969	7	146526969	4	-0.320728183	G/T	4.60E-22
PZE07146806148	7	146806148	79	-0.324339829	C/G	8.20E-10
PZE07146820075	7	146820075	9	-0.294735591	G/A	6.18E-14
PZE07147573753	7	147573753	2	-0.411362852	A/G	4.77E-13
PZE07147613301	7	147613301	5	0.520757899	G/A	6.09E-08
PZE07149386177	7	149386177	2	0.244555179	T/C	8.92E-09
PZE07149386215	7	149386215	2	0.261288508	A/C	1.46E-07
PZE07149387396	7	149387396	2	0.248149514	G/T	1.17E-07
PZE07151502735	7	151502735	2	0.225011243	A/C	2.69E-07
PZE07151989475	7	151989475	3	0.400636152	G/T	1.67E-07
PZE07166483247	7	166483247	6	0.360385703	T/C	1.31E-08
PZE07167726305	7	167726305	6	0.207284606	C/A	1.09E-08
PZE07167730525	7	167730525	4	0.190570049	C/G	3.96E-09
PZE07167772571	7	167772571	3	0.252309051	-/C	2.09E-08
PZE07168145531	7	168145531	2	0.17235345	A/T	1.46E-09
PZE07168146930	7	168146930	7	0.173593493	A/G	4.49E-09
PZE07168743114	7	168743114	27	0.17193935	G/C	8.43E-09
PZE07168976547	7	168976547	4	0.162396727	------/GTATAA	8.28E-09
PZE07168984213	7	168984213	8	0.250917441	G/T	2.02E-08
PZE07168992020	7	168992020	2	0.224958116	G/C	1.90E-09
PZE07168992525	7	168992525	3	0.17221707	C/A	1.20E-09
PZE07169183797	7	169183797	9	0.155247095	T/C	6.73E-09
PZE07169419976	7	169419976	3	0.152305871	A/C	1.53E-08
PZE07169531151	7	169531151	2	0.191372002	C/A	6.10E-11
PZE07169769954	7	169769954	2	0.267798468	GA/--	1.73E-07
PZE07169955290	7	169955290	2	0.152358815	G/C	1.67E-08
PZE0801509107	8	1509107	4	-0.162082771	C/T	2.48E-07
PZE0804839712	8	4839712	2	-0.196336697	T/C	9.49E-08
PZE0805757503	8	5757503	2	-0.181529636	G/A	1.82E-09

PZE0806188833	8	6188833	4	-0.206035585	G/C	9.95E-09
PZE0806803581	8	6803581	3	-0.184040174	C/T	1.62E-07
PZE0806989622	8	6989622	2	-0.152768038	T/G	1.51E-09
PZE0807385190	8	7385190	2	-0.193616961	T/C	4.87E-08
PZE0807570909	8	7570909	23	-0.169489296	C/T	2.11E-08
PZE0807570941	8	7570941	2	-0.17212456	T/C	1.27E-07
PZE0807864759	8	7864759	2	-0.15056144	A/C	1.71E-08
PZE0810036998	8	10036998	29	-0.182525859	---/TGT	2.13E-08
PZE0810195404	8	10195404	2	-0.189277261	A/T	8.08E-09
PZE0865717661	8	65717661	2	-0.22804937	A/G	7.69E-08
PZE0868342924	8	68342924	5	-0.195083766	A/G	1.63E-07
PZE0868838442	8	68838442	2	-0.197072244	G/A	4.93E-10
PZE0868910083	8	68910083	5	-0.181507519	T/C	1.22E-08
PZE0870537580	8	70537580	2	-0.195868721	T/C	1.58E-10
PZE0895053302	8	95053302	15	-0.226360082	G/C	1.58E-08
PZE0895491931	8	95491931	2	-0.221493703	A/G	2.04E-11
PZE0895553351	8	95553351	7	-0.19530324	T/C	6.93E-08
PZE08100012904	8	100012904	2	-0.202545331	G/C	2.26E-07
PZE08107163817	8	107163817	10	-0.227665439	G/T	9.56E-08
PZE08108910314	8	108910314	2	-0.224190216	---/CTC	4.47E-07
PZE08112215505	8	112215505	2	-0.355357321	A/G	9.79E-08
PZE08119131096	8	119131096	5	-0.6747724	C/G	2.76E-13
PZE08120511414	8	120511414	67	0.397544107	T/G	3.97E-11
PZE08120511453	8	120511453	3	0.263231299	G/T	2.37E-08
PZE08120511504	8	120511504	7	0.427589648	T/G	1.31E-08
PZE08121630271	8	121630271	9	-0.541083067	C/T	5.78E-14
PZE08122169246	8	122169246	2	0.421633063	G/C	7.70E-08
PZE08123914777	8	123914777	5	-0.550183027	G/T	2.93E-14
PZE08124586510	8	124586510	6	0.332889258	A/T	6.76E-11
PZE08130203010	8	130203010	2	0.304612512	T/C	1.26E-15
PZE08130210053	8	130210053	2	0.265307519	-/A	6.37E-05
PZE08130213299	8	130213299	6	0.300698003	T/G	2.70E-09
PZE08130215474	8	130215474	7	0.32007091	G/A	5.34E-07
PZE08130662170	8	130662170	4	-0.51767767	--/CG	3.40E-14
PZE08130918693	8	130918693	2	-0.37946911	A/G	2.83E-12
PZE08130986937	8	130986937	65	-0.60689554	C/A	1.01E-12
PZE08131060815	8	131060815	6	-0.594328543	T/C	1.40E-15
PZE08134135651	8	134135651	2	0.273998162	---/TCA	1.75E-07
PZE08144674143	8	144674143	5	0.215720909	T/C	1.67E-07
PZE08144679698	8	144679698	2	0.208224704	----/GGGG	4.55E-08
PZE08148169111	8	148169111	2	0.214397693	A/G	3.14E-07
PZE08150605029	8	150605029	2	0.198327232	T/G	3.32E-07
PZE08151445933	8	151445933	2	0.353661082	C/G	3.12E-07
PZE08155135524	8	155135524	2	0.216625652	G/A	3.20E-07
PZE08160241331	8	160241331	5	0.343612336	G/C	2.84E-10
PZE08161241598	8	161241598	2	0.244909437	T/C	8.46E-11
PZE08161249047	8	161249047	48	0.282613177	---/CAG	1.58E-08
PZE08161266547	8	161266547	14	0.290478926	-/G	1.56E-14
PZE08161266696	8	161266696	7	0.239034798	-/T	2.84E-09

PZE08161918620	8	161918620	4	0.223269384	G/A	2.16E-09
PZE08162433794	8	162433794	2	0.274811639	CG/--	6.13E-11
PZE08162579032	8	162579032	2	0.227149458	C/T	1.55E-10
PZE08162713717	8	162713717	2	0.270952361	G/A	9.85E-14
PZE08162714483	8	162714483	6	0.238036655	G/C	1.01E-10
PZE08168925295	8	168925295	4	0.458360527	G/C	4.73E-07
PZE0906546871	9	6546871	4	0.238196314	G/A	1.64E-07
PZE0918206377	9	18206377	5	0.214516017	A/C	3.19E-08
PZE0918786666	9	18786666	10	0.219844073	A/T	1.48E-07
PZE0925069281	9	25069281	6	-0.556048745	G/T	1.92E-08
PZE0925219444	9	25219444	5	-0.346460044	C/G	9.82E-11
PZE0925305327	9	25305327	58	-0.441398538	G/C	1.36E-07
PZE0925421687	9	25421687	6	0.54485485	A/G	2.09E-07
PZE0931580222	9	31580222	2	0.815277449	G/A	1.27E-10
PZE0940769299	9	40769299	2	-0.362921941	C/A	5.07E-11
PZE0944360733	9	44360733	3	-0.462399841	C/T	1.02E-12
PZE0955166685	9	55166685	6	-0.490558938	C/T	2.00E-09
PZE0961378545	9	61378545	4	-0.294355164	A/G	1.43E-09
PZE0979783823	9	79783823	11	-0.395048535	T/C	4.84E-09
PZE0987771126	9	87771126	2	0.359265239	A/T	4.28E-08
PZE0987772588	9	87772588	2	0.317356811	T/C	4.72E-08
PZE0993250419	9	93250419	3	0.521983098	T/C	1.67E-10
PZE09102126133	9	102126133	3	0.377115039	C/T	1.36E-11
PZE09114695850	9	114695850	3	0.270069304	C/G	1.35E-08
PZE09114697012	9	114697012	2	0.515341825	G/T	4.91E-09
PZE09115198367	9	115198367	73	0.65810409	C/G	6.28E-14
PZE09116457796	9	116457796	2	0.528655701	G/A	2.29E-08
PZE09117144529	9	117144529	2	0.433594678	G/A	3.96E-07
PZE09118281318	9	118281318	2	0.354417084	T/C	2.85E-07
PZE09118686452	9	118686452	26	0.514091535	T/C	4.92E-08
PZE09119041104	9	119041104	3	0.254601965	A/G	8.20E-09
PZE09119910643	9	119910643	3	0.396329095	A/G	0.00048855
PZE09120246827	9	120246827	5	0.477165949	C/T	8.17E-12
PZE09121443695	9	121443695	2	0.324062537	G/A	7.13E-07
PZE09122155651	9	122155651	32	0.350589533	G/C	7.88E-08
PZE09122944989	9	122944989	4	0.459998593	A/T	1.50E-09
PZE09123345708	9	123345708	7	0.295007423	C/T	1.22E-11
PZE09123346148	9	123346148	3	0.300004086	A/G	1.80E-15
PZE09124303569	9	124303569	2	0.300654962	GA/--	4.71E-08
PZE09124602918	9	124602918	14	0.273316204	T/G	1.39E-08
PZE09124603238	9	124603238	2	0.223309209	--/GA	8.24E-09
PZE09129254134	9	129254134	56	0.321006925	-------/ACCAGTA	6.45E-11
PZE09131490476	9	131490476	2	0.207131758	T/A	9.51E-08
PZE09133803648	9	133803648	3	0.29461902	A/G	1.75E-09
PZE09138740689	9	138740689	3	-0.349269777	A/G	2.55E-07
PZE09138755179	9	138755179	4	-0.351459696	T/C	3.73E-07
PZE09140383003	9	140383003	2	-0.262671476	---/GAA	4.76E-07
PZE09147680069	9	147680069	2	-0.193859175	T/A	2.69E-07
PZE1004218759	10	4218759	3	0.358300259	A/C	5.23E-07

PZE1004980095	10	4980095	2	0.156406978	A/G	4.17E-07
PZE1004985862	10	4985862	2	0.152961572	C/T	5.38E-07
PZE1005598937	10	5598937	7	0.193022713	G/A	1.24E-07
PZE1005926447	10	5926447	2	0.227801786	A/G	3.03E-08
PZE1005929742	10	5929742	3	0.187777457	T/C	1.29E-07
PZE1006069292	10	6069292	44	0.227768762	G/-	5.25E-08
PZE1008265748	10	8265748	2	0.344215822	A/G	3.01E-08
PZE1008763030	10	8763030	2	0.348032778	G/C	9.13E-08
PZE1009836886	10	9836886	17	0.232424535	A/-	3.23E-08
PZE1081633642	10	81633642	2	-0.311421443	G/A	1.68E-09
PZE1089281203	10	89281203	4	0.656386115	G/A	3.37E-08
PZE1091592554	10	91592554	11	0.668679667	A/G	1.61E-07
PZE1091770977	10	91770977	20	-0.332429694	T/C	1.85E-08
PZE1094732989	10	94732989	2	-0.543618453	G/A	2.96E-12
PZE10102590312	10	102590312	5	1.095551217	G/T	6.57E-13
PZE10109543539	10	109543539	92	1.068053051	G/T	4.30E-10
PZE10128858354	10	128858354	52	-0.309600467	--/CA	2.68E-08
PZE10132184474	10	132184474	4	-0.354905024	G/A	7.16E-08
PZE10142350903	10	142350903	2	0.403049398	C/G	2.16E-07
PZE10142352026	10	142352026	5	0.483796163	G/T	3.74E-07
PZE10143342970	10	143342970	5	-0.223007002	G/C	7.66E-10
PZE10144241771	10	144241771	2	-0.194409248	------/TAGGAA	8.91E-09
PZE10144834789	10	144834789	4	-0.238968252	---/CTA	8.78E-12
PZE10144836415	10	144836415	3	-0.234247724	C/G	1.02E-10
PZE10145504856	10	145504856	2	0.332466498	T/-	6.11E-08
PZE10146134080	10	146134080	25	-0.270498743	G/A	1.21E-10
PZE10146709001	10	146709001	16	-0.293778365	C/A	4.65E-10
PZE10146709011	10	146709011	7	-0.259739783	G/A	5.01E-11
PZE10146709163	10	146709163	10	-0.296554892	G/T	2.01E-09
PZE10147004826	10	147004826	13	0.232615313	C/T	2.65E-07
PZE10147004835	10	147004835	2	0.244255003	A/G	3.72E-07
PZE10147140324	10	147140324	15	-0.322225891	T/-	2.96E-08
PZE10147140332	10	147140332	2	-0.385343294	C/T	2.56E-10
PZE10147395829	10	147395829	2	0.416965343	-/T	2.19E-07
PZE10147396963	10	147396963	3	0.2171616	C/T	5.20E-07
PZE10147441332	10	147441332	2	0.301775547	G/A	4.78E-07
PZE10147441641	10	147441641	2	-0.38662307	--/AC	3.93E-09
PZE10147983838	10	147983838	4	0.221956719	T/G	9.43E-08
PZE10147984352	10	147984352	10	0.25509069	G/A	2.32E-07
