# Supplementary material for: PICARA, an Analytical Pipeline Providing Probabilistic Inference about A Priori Candidates Genes Underlying Genome-Wide Association QTL in Plants
Source: PLoS One. 2012 Nov 7;7(11):e46596. doi: 10.1371/journal.pone.0046596 (PMC3492367; doi:10.1371/journal.pone.0046596)
Supplement: Table S3 — Maize flowering time priori candidates and the annotation from their Arabidopsis homologs. (PDF) [file pone.0046596.s004.pdf]

Table S3. Maize flowering time priori candidates and the annotation from their *Arabidopsis* homologs

chr	gene.id	start	end	Compara score	linkage block window estimate	SNP count	GWAS count	LOD	annotation from *Arabidopsis*
1	GRMZM2G180909	94872408	94875867	2	whole chr. estimate*	147	2	4.70	FLK; PEP
1	GRMZM2G114184	21720856	21722289	1	whole chr. estimate*	454	3	4.42	HLS1
1	GRMZM2G379656	234417882	234419889	2	whole chr. estimate*	75	3	2.67	PCL1; LUX; AT5G59570
2	GRMZM2G147241	29664742	29666631	2	136660	94	2	5.22	AT3G04510; LSH1
2	GRMZM2G061734	189216581	189221629	4	82562	114	2	4.83	SPL3,4,5,9
2	GRMZM2G351330	21546030	21547337	2	5980	133	2	4.52	HAT4; ATHB51
2	GRMZM2G059358	68550222	68551988	6	whole chr. estimate*	182	2	3.89	MFT; FT; ATC; TSF; TFL1; BFT
2	GRMZM2G033962	215747350	215752804	5	32339	54	1	2.81	APRR3,5,7,9; TOC1
2	GRMZM2G080054	9400025	9401900	7	48301	57	1	2.75	HFR1; PAP3; PIF3,4,6,7; PIL2,5,6
2	GRMZM2G422644	192253932	192261552	1	128291	72	1	2.52	STO
2	GRMZM2G098859	208269128	208278594	1	103026	82	1	2.39	CIP7
2	GRMZM2G143602	233162053	233167522	4	32290	83	1	2.38	CK2A/CK2alphaC; CKA2/CK2alphaB; CKA1
2	GRMZM2G378106	206167242	206169823	2	3919	92	1	2.28	DFL1, 2
2	GRMZM2G026309	200624002	200632168	1	66555	104	1	2.15	TOR1
2	GRMZM2G107945	221889489	221893852	3	26935	118	1	2.03	FKF1; LKP2; ZTL
3	GRMZM2G115960	48304981	48308663	7	141762	124	6	19.03	HFR1; PAP3; PIF3,4,6,7; PIL2,5,6
3	GRMZM2G365688	222026943	222033445	5	whole chr. estimate*	317	3	5.12	APRR3, 5, 7, 9; TOC1
3	GRMZM2G103666	195839573	195841459	6	46176	20	1	3.87	MFT; FT; ATC; TSF; TFL1; BFT
3	GRMZM2G024851	135105040	135109074	1	213828	61	1	2.75	VIP1
3	GRMZM2G180406	160801253	160804532	1	3832	93	1	2.33	CIB1
3	GRMZM2G026643	27197008	27204698	2	26699	105	1	2.21	PDF2; FWA
3	GRMZM2G437460	208704105	208712275	1	92648	106	1	2.20	NPH4
3	GRMZM2G126239	213282365	213286757	2	8876	117	1	2.10	HAT4; ATHB51
3	GRMZM2G067511	38095885	38100475	1	186014	119	1	2.08	CAM4
3	GRMZM2G178102	119446822	119453639	2	227634	123	1	2.05	PDF2; FWA
3	GRMZM2G466139	204589152	204607437	1	202861	126	1	2.03	ESP4
4	GRMZM2G120167	16093643	16098788	1	whole chr. estimate*	165	2	4.67	VIP1
4	GRMZM2G362857	166063496	166064228	1	37897	63	1	2.95	TCH2

4	GRMZM2G060464	119982155	119984821	1	257256	115	1	2.34	NDPK2
4	GRMZM2G001048	14693823	14697226	2	113197	134	1	2.19	RTV1; VRN1
5	GRMZM2G176688	14494964	14499875	1	19534	71	2	6.40	PHP; CDC73
5	GRMZM2G012302	165484008	165491930	1	141669	57	1	3.06	TEL1
5	GRMZM2G479110	212929345	212934149	2	14439	60	1	3.01	PCL1; LUX; AT5G59570
5	GRMZM2G138421	1849506	1856495	4	22109	61	1	2.99	SPL3, 4, 5, 9
5	GRMZM2G146354	73575645	73578793	1	146545	78	1	2.75	SINAT5
5	GRMZM2G029850	196753590	196756281	3	16988	104	1	2.46	LHY; CCA1; LCL1
5	GRMZM2G030013	72326234	72331866	2	80683	108	1	2.42	FLK; PEP
5	GRMZM2G160565	195716989	195724582	20	37141	118	1	2.33	SEPALLATA1, 2, 3, 4; CAL; SHP1,2, SOC1, AP1; AG; AGL6,8,13,14,16,17,19,21,79; STK; FUL
5	GRMZM2G041991	207119148	207121191	7	21798	121	1	2.31	CO; COL1,2,3,9; AtCOL4; ATCOL5
6	GRMZM2G366778	165108382	165109343	9	whole chr. estimate*	212	2	4.29	GRF1,2,3,4,5,6,7,8,9;
6	GRMZM2G032332	13567016	13578512	1	159996	92	1	2.63	KNAT5
7	GRMZM2G086656	169601264	169608797	1	whole chr. estimate*	119	2	4.80	CR88
7	GRMZM2G179024	137538236	137543869	5	9710	29	1	3.46	APRR3, 5, 7, 9; TOC1
7	GRMZM2G005732	169183308	169216864	5	164032	83	1	2.41	APRR3, 5, 7, 9; TOC1
7	GRMZM2G034288	40409096	40422236	2	404401	96	1	2.26	ASHH1; SDG26; EFS
7	GRMZM2G013794	61327400	61348210	4	433945	99	1	2.23	CLF; ATX1; EZA1; SWN1; SDG25
7	GRMZM2G024477	61303282	61325847	4	441186	100	1	2.22	CLF; ATX1; EZA1; SWN1; SDG25
7	AC233869.1_FG003	124221415	124223557	1	4335	110	1	2.13	CIB1
8	GRMZM2G098443	95491013	95491879	6	whole chr. estimate*	74	3	9.51	TEM1,2; RAVL1,2,3; ARF14
8	GRMZM2G030762	161261193	161264349	1	14560	152	2	4.37	CIB1
8	GRMZM2G375448	162367676	162369010	10	whole chr. estimate*	401	2	2.42	NF-YC1,2,3,4,5,6,8,9,12
8	GRMZM2G155216	69073505	69075667	2	406335	99	1	2.26	AT5G28450; CAB1A; LHCB*3
8	GRMZM2G363429	134136139	134138436	2	41215	99	1	2.26	BR6OX2; CPD
9	GRMZM2G126120	115386801	115392016	2	84466	78	2	6.10	FLK; PEP
9	GRMZM2G148969	122948899	122951228	1	10062	100	1	2.44	BAS1
9	GRMZM2G407119	140385843	140389111	1	47752	143	1	2.09	CIB1
10	GRMZM2G097289	89121551	89126741	1	whole chr. estimate*	57	2	6.75	SYD
10	GRMZM2G093895	9840578	9842684	1	5188	66	1	2.87	CHE
10	AC207755.3_FG006	5597566	5598563	6	1707	76	1	2.73	TEM1,2; RAVL1,2,3; ARF14

10	GRMZM2G062541	87614256	87616290	7		240274	109	1	2.37	HFR1; PAP3; PIF3,4,6,7; PIL2,5,6
10	GRMZM2G042920	142353602	142355628	7		5281	111	1	2.35	HFR1; PAP3; PIF3,4,6,7; PIL2,5,6

* whole chromosome estimate of linkage block window sizes are provided to the enrichment analysis when genes are in SNP poor region and the local estimate are not available
