# Supplementary material for: PICARA, an Analytical Pipeline Providing Probabilistic Inference about A Priori Candidates Genes Underlying Genome-Wide Association QTL in Plants
Source: PLoS One. 2012 Nov 7;7(11):e46596. doi: 10.1371/journal.pone.0046596 (PMC3492367; doi:10.1371/journal.pone.0046596)
Supplement: Table S4 — GO analysis of maize flowering time priori candidate genes. (PDF) [file pone.0046596.s005.pdf]

Table S4. GO analysis of maize flowering time priori candidate genes.

GO term	category	description	associated	bgitem	bgtotal	pvalue	FDR
GO:0045449	Biological process	regulation of transcription	24	657	13162	1.10E-14	1.50E-12
GO:0019219	Biological process	regulation of nucleobase, nucleoside, nucleotide and nucleic acid metabolic process	24	692	13162	3.20E-14	1.50E-12
GO:0051171	Biological process	regulation of nitrogen compound metabolic process	24	692	13162	3.20E-14	1.50E-12
GO:0006350	Biological process	transcription	24	717	13162	6.80E-14	2.30E-12
GO:0031326	Biological process	regulation of cellular biosynthetic process	24	746	13162	1.50E-13	3.00E-12
GO:0009889	Biological process	regulation of biosynthetic process	24	746	13162	1.50E-13	3.00E-12
GO:0010556	Biological process	regulation of macromolecule biosynthetic process	24	743	13162	1.40E-13	3.00E-12
GO:0010468	Biological process	regulation of gene expression	24	775	13162	3.40E-13	5.70E-12
GO:0031323	Biological process	regulation of cellular metabolic process	24	808	13162	7.90E-13	1.20E-11
GO:0080090	Biological process	regulation of primary metabolic process	24	819	13162	1.00E-12	1.40E-11
GO:0060255	Biological process	regulation of macromolecule metabolic process	24	840	13162	1.70E-12	2.10E-11
GO:0019222	Biological process	regulation of metabolic process	24	870	13162	3.50E-12	3.90E-11
GO:0006355	Biological process	regulation of transcription, DNA-dependent	16	419	13162	6.40E-11	6.60E-10
GO:0051252	Biological process	regulation of RNA metabolic process	16	446	13162	1.60E-10	1.50E-09
GO:0032774	Biological process	RNA biosynthetic process	16	461	13162	2.50E-10	2.10E-09
GO:0006351	Biological process	transcription, DNA-dependent	16	461	13162	2.50E-10	2.10E-09
GO:0006139	Biological process	nucleobase, nucleoside, nucleotide and nucleic acid metabolic process	26	1373	13162	1.40E-09	1.10E-08
GO:0050794	Biological process	regulation of cellular process	25	1317	13162	2.40E-09	1.80E-08
GO:0050789	Biological process	regulation of biological process	25	1421	13162	9.80E-09	6.90E-08
GO:0065007	Biological process	biological regulation	26	1540	13162	1.20E-08	8.20E-08
GO:0034645	Biological process	cellular macromolecule biosynthetic process	24	1392	13162	2.40E-08	1.60E-07
GO:0010467	Biological process	gene expression	24	1403	13162	2.80E-08	1.70E-07
GO:0016070	Biological process	RNA metabolic process	16	693	13162	6.70E-08	3.90E-07
GO:0009059	Biological process	macromolecule biosynthetic process	24	1489	13162	7.90E-08	4.40E-07
GO:0006807	Biological process	nitrogen compound metabolic process	26	2050	13162	1.90E-06	1.00E-05
GO:0044249	Biological process	cellular biosynthetic process	25	2109	13162	7.90E-06	4.10E-05
GO:0009058	Biological process	biosynthetic process	25	2226	13162	1.80E-05	8.90E-05
GO:0044260	Biological process	cellular macromolecule metabolic process	27	2760	13162	8.20E-05	0.0004
GO:0043170	Biological process	macromolecule metabolic process	28	3132	13162	0.00024	0.0011
GO:0044238	Biological process	primary metabolic process	29	4014	13162	0.0034	0.015

GO:0044237	Biological process	cellular metabolic process	29	4007	13162	0.0033	0.015
GO:0008152	Biological process	metabolic process	30	4683	13162	0.012	0.05
GO:0009987	Biological process	cellular process	31	5051	13162	0.017	0.071
GO:0030528	Molecular function	transcription regulator activity	19	400	13162	3.60E-14	1.40E-12
GO:0043565	Molecular function	sequence-specific DNA binding	9	130	13162	3.70E-09	7.00E-08
GO:0003677	Molecular function	DNA binding	18	752	13162	7.50E-09	9.30E-08
GO:0003676	Molecular function	nucleic acid binding	22	1382	13162	2.70E-07	2.50E-06
GO:0003700	Molecular function	transcription factor activity	8	285	13162	2.20E-05	0.00016
GO:0005488	Molecular function	binding	37	4937	13162	0.0009	0.0056
GO:0008270	Molecular function	zinc ion binding	6	585	13162	0.026	0.12
GO:0046872	Molecular function	metal ion binding	10	1276	13162	0.029	0.12
GO:0046914	Molecular function	transition metal ion binding	8	923	13162	0.029	0.12
GO:0043169	Molecular function	cation binding	10	1331	13162	0.037	0.13
GO:0043167	Molecular function	ion binding	10	1336	13162	0.038	0.13
GO:0003824	Molecular function	catalytic activity	8	4121	13162	0.97	1
GO:0005634	Cellular component	nucleus	20	1146	13162	2.00E-07	5.10E-06
GO:0043226	Cellular component	organelle	21	5850	13162	0.57	1
GO:0043229	Cellular component	intracellular organelle	21	5848	13162	0.57	1
GO:0005622	Cellular component	intracellular	25	6442	13162	0.44	1
GO:0043227	Cellular component	membrane-bounded organelle	21	5453	13162	0.46	1
GO:0005623	Cellular component	cell	27	7508	13162	0.57	1
GO:0043231	Cellular component	intracellular membrane-bounded organelle	21	5449	13162	0.46	1
GO:0044464	Cellular component	cell part	27	7508	13162	0.57	1
GO:0044424	Cellular component	intracellular part	21	6346	13162	0.69	1
