# Supplementary material for: PICARA, an Analytical Pipeline Providing Probabilistic Inference about A Priori Candidates Genes Underlying Genome-Wide Association QTL in Plants
Source: PLoS One. 2012 Nov 7;7(11):e46596. doi: 10.1371/journal.pone.0046596 (PMC3492367; doi:10.1371/journal.pone.0046596)
Supplement: Table S5 — Arabidopsis gene IDs for GO terms. (PDF) [file pone.0046596.s006.pdf]

Table S5. *Arabidopsis* gene IDs for GO terms.

GO: 0009058	GO:0032502	GO:0030234	GO:0040007	GO:0048585	GO: 0048584	GO:0030528
AT5G59070	AT5G58440	AT1G54040	AT1G17420	AT5G20910	AT5G51450	AT5G25150
AT1G04920	AT2G42870	AT1G15700	AT3G53760	AT3G51060	AT3G55530	AT2G22300
AT1G16570	AT3G55240	AT2G20580	AT5G43630	AT3G52400	AT2G22630	AT5G09850
AT1G03210	AT3G45890	AT4G28470	AT1G73030	AT5G08335	AT1G09665	AT5G63160
AT1G63660	AT3G58850	AT4G04640	AT4G33950	AT5G25350	AT1G09770	AT5G17560
AT4G02850	AT3G57180	AT2G32730	AT1G55020	AT4G12560	AT2G45130	AT3G28857
AT4G01210	AT1G23540	AT1G20200	AT2G18250	AT1G64280	AT1G56160	AT1G19050
AT1G32900	AT1G26910	AT1G04810	AT5G47040	AT3G48040	AT1G19250	AT3G47610
AT4G17360	AT2G30280	AT2G39730	AT1G15510	AT5G64750	AT2G33150	AT5G64340
AT1G74910	AT5G07120	AT1G75990	AT1G29410	AT5G63980	AT1G22770	AT1G44810
AT1G62730	AT1G26210	AT4G16500	AT4G31700	AT1G66340	AT3G63010	AT4G35540
AT1G74010	AT3G10400		AT5G10360	AT3G23150	AT3G15730	AT2G44120
AT5G40870	AT5G53400		AT1G75840	AT3G61190	AT3G05120	AT2G46790
AT3G48790	AT3G53900		AT5G57020	AT5G65050	AT1G35670	AT4G25520
AT2G18250	AT2G30490		AT1G67560	AT4G10180	AT5G18200	AT2G41310
AT2G04650	AT1G68870		AT1G17730	AT4G35390	AT4G19530	AT5G62920
AT2G32260	AT1G33410		AT2G30490	AT4G24580	AT3G50500	AT2G37678
AT2G38670	AT1G80680		AT1G72520	AT3G25070	AT3G25070	AT4G16265
AT4G02860			AT3G26940	AT1G69935	AT1G25490	AT4G25210
AT4G15130			AT3G43300	AT2G39550	AT4G31120	AT1G80750
AT5G55810			AT1G77740	AT1G50640	AT2G33835	AT1G61730
AT3G55870				AT1G22920	AT5G20150	AT4G00270
AT1G77130				AT3G04580	AT3G05360	AT1G30455
AT1G25155				AT5G57050	AT3G56400	AT1G09530
AT1G72330				AT5G46210	AT4G03110	AT5G11530
AT2G41300				AT3G07650	AT1G25540	AT2G17390
AT5G36160				AT5G42970	AT3G18165	AT5G35770
AT3G55590				AT2G25170	AT5G45250	AT5G20730
AT2G04540				AT3G03450	AT1G08860	AT5G18230
AT1G19710				AT2G22430	AT4G12720	AT2G36340
AT5G20280				AT4G18470	AT3G18690	AT3G13580
AT1G74000				AT5G01810	AT2G04450	AT3G20740
AT2G13810				AT4G39030	AT2G31880	AT3G10820
AT4G23600				AT2G20180	AT2G43790	AT4G31900
AT4G26510				AT5G09230	AT4G15900	AT5G13240
AT1G63680				AT4G05420	AT1G08450	AT4G01260
AT4G00040				AT5G03280	AT5G40440	AT4G10680
AT4G19460				AT1G14920	AT2G17090	AT1G35515
AT1G54940				AT3G11820	AT5G59890	AT5G15840
AT4G10120				AT3G52430	AT4G03550	AT4G18890
AT1G73980				AT2G25490	AT3G25040	AT3G23980
AT4G18300				AT2G26070	AT3G57300	AT3G16940
AT1G62800				AT1G08260	AT5G47220	AT4G10920
AT3G15940				AT1G18075	AT5G03280	AT1G75080
AT1G77670				AT1G01720	AT5G66880	AT5G59710
AT3G57010				AT4G26080	AT2G32460	AT5G64220

AT2G39630	AT1G77470	AT1G33520	AT2G20100
AT2G39770	AT1G71230	AT2G26300	AT2G41630
AT1G08990	AT3G11410	AT5G27320	AT2G13690
AT2G36690	AT1G74710	AT5G06100	AT4G00250
AT3G01180	AT5G40280	AT1G80680	AT4G25515
AT4G23590	AT2G46340	AT4G09570	AT5G05140
AT5G22020	AT3G59380	AT5G61900	AT1G07705
AT5G18480	AT4G36260	AT4G23510	AT1G49830
AT3G57020	AT2G01570	AT1G04450	AT1G29950
AT4G08040	AT2G39940	AT4G25230	AT1G10586
AT4G16600	AT3G19140	AT3G15210	AT1G68210
AT5G60700	AT3G15210	AT4G24210	AT1G51450
AT2G34970	AT5G03730	AT4G23450	AT2G40670
AT1G25083	AT2G40940	AT5G53160	AT1G01210
AT2G01220	AT3G02140	AT2G46370	AT4G00390
AT3G60290	AT2G46370	AT5G60410	AT2G27050
AT1G55810	AT3G11540	AT5G20480	AT1G67310
AT5G04620	AT5G23320	AT1G71220	AT2G46020
AT3G07270	AT1G66350	AT1G15100	AT1G10470
AT3G57030	AT1G59870	AT5G40990	AT5G09460
AT5G19220	AT5G48380		AT3G50750
AT3G27190	AT5G04240		AT5G24470
AT2G12200	AT1G11310		AT3G29380
AT5G19485	AT1G73687		AT2G45190
AT1G66520	AT1G04310		AT4G26500
AT2G24850	AT4G37460		AT1G11510
AT1G78800			AT3G04280
AT1G26190			AT2G17950
AT2G12280			AT1G27045
AT1G24909			AT2G20570
AT1G73160			AT1G55805
AT3G02270			AT1G59940
AT5G11110			AT5G67480
AT5G66120			AT5G44190
AT1G03030			AT3G56380
AT1G52420			AT2G44980
AT1G05610			AT3G48360
AT5G04480			AT4G28811
AT3G10340			AT2G25650
AT3G27440			AT4G00730
AT1G32060			AT4G19550
AT2G01460			AT3G57370
AT1G24807			AT5G62380
AT3G45100			AT1G78700
AT5G47435			AT3G09360
AT3G51420			AT5G02810
AT1G75420			AT5G60100
AT3G51450			AT5G50010
AT4G28410			AT5G39230

AT5G13630	AT5G14280
AT2G41290	AT4G28815
AT2G35710	AT3G04930
AT3G10630	AT4G36780
AT3G18660	AT1G26945
AT4G33330	AT3G56220
AT1G80360	AT3G16980
AT1G73740	AT1G66420
AT1G31220	AT2G47270
AT5G03770	AT3G57040
AT3G51440	AT4G00760
AT4G16710	AT1G02080
AT4G30570	AT3G25940
AT1G08470	AT5G14750
AT5G53970	AT5G09830
AT4G28420	AT1G74890
	AT2G45640
	AT5G62120
	AT4G37610
	AT1G79430
	AT5G09410
	AT1G71930
	AT1G29220
	AT1G19350
	AT4G30180
	AT4G00610
	AT2G45100
	AT1G05690
	AT3G48050
	AT5G61380
	AT4G07950
	AT3G10330
	AT3G48060
	AT2G01250
	AT3G48100
	AT5G28040
	AT2G01370
	AT4G16150
